# Membrane compression by synaptic vesicle exocytosis triggers ultrafast endocytosis

**DOI:** 10.1038/s41467-023-38595-2

**Published:** 2023-05-20

**Authors:** Tyler H. Ogunmowo, Haoyuan Jing, Sumana Raychaudhuri, Grant F. Kusick, Yuuta Imoto, Shuo Li, Kie Itoh, Ye Ma, Haani Jafri, Matthew B. Dalva, Edwin R. Chapman, Taekjip Ha, Shigeki Watanabe, Jian Liu

**Affiliations:** 1 Department of Cell Biology, School of Medicine, Johns Hopkins University, Baltimore, MD US; 2 Center for Cell Dynamics, School of Medicine, Johns Hopkins University, Baltimore, MD US; 3 Biochemistry, Cellular and Molecular Biology graduate program, Johns Hopkins University, Baltimore, MD US; 4 Department of Biomedical Engineering, School of Medicine, Johns Hopkins University, Baltimore, MD US; 5 Department of Neuroscience and Jefferson Synaptic Biology Center, Thomas Jefferson University, Philadelphia, PA US; 6 Department of Neuroscience, University of Wisconsin-Madison, Madison, WI US; 7 Howard Hughes Medical Institute, Madison, WI US; 8 Department of Biophysics and Biophysical Chemistry, School of Medicine, Johns Hopkins University, Baltimore, MD US; 9 Department of Biophysics, Johns Hopkins University, Baltimore, MD US; 10 Howard Hughes Medical Institute, Baltimore, MD US; 11 Solomon H. Snyder Department of Neuroscience, School of Medicine, Johns Hopkins University, Baltimore, MD US; 12 Present Address: Department of Ophthalmology, School of Medicine, Stanford University, Palo Alto, CA US; 13 Present Address: Department of Cell and Molecular Biology and the Tulane Brain Institute, Tulane University, New Orleans, LA US

**Keywords:** Endocytosis, Membrane curvature, Endocytosis, Exocytosis, Synaptic vesicle endocytosis

## Abstract

Compensatory endocytosis keeps the membrane surface area of secretory cells constant following exocytosis. At chemical synapses, clathrin-independent ultrafast endocytosis maintains such homeostasis. This endocytic pathway is temporally and spatially coupled to exocytosis; it initiates within 50 ms at the region immediately next to the active zone where vesicles fuse. However, the coupling mechanism is unknown. Here, we demonstrate that filamentous actin is organized as a ring, surrounding the active zone at mouse hippocampal synapses. Assuming the membrane area conservation is due to this actin ring, our theoretical model suggests that flattening of fused vesicles exerts lateral compression in the plasma membrane, resulting in rapid formation of endocytic pits at the border between the active zone and the surrounding actin-enriched region. Consistent with model predictions, our data show that ultrafast endocytosis requires sufficient compression by exocytosis of multiple vesicles and does not initiate when actin organization is disrupted, either pharmacologically or by ablation of the actin-binding protein Epsin1. Our work suggests that membrane mechanics underlie the rapid coupling of exocytosis to endocytosis at synapses.

## Abstract (web summary)

The authors describe the mechanism of exo-endocytosis coupling at synapses. They find that actin forms a ring around the region of exocytosis. This ring conserves membrane area, allowing induction of inward membrane buckling following exocytosis.

## Introduction

Neurons communicate with target cells through release of chemical signals at intimate contact sites called synapses. In the communicating neuron’s side of the synapse, the presynapse, there are a few hundred synaptic vesicles that contain chemical signals, or neurotransmitters. Upon neuronal stimulation, some of these vesicles undergo exocytosis in a region defined as the active zone, which is directly opposed to the postsynaptic membrane. After exocytosis, vesicle membrane must be retrieved from the plasma membrane through endocytosis to keep the area of the plasma membrane constant and maintain a sufficient number of synaptic vesicles at presynapses^1–4^.

Several recent studies suggest that synaptic vesicle endocytosis is mediated by clathrin-independent ultrafast endocytosis at mammalian central synapses and is tightly coupled to exocytosis in time and space^5–9^. Indeed, ultrafast endocytosis initiates as fast as 20–50 ms after exocytosis and completes within 100 ms^5–7^. In this pathway, endocytic invaginations always form at the edge of the active zone^5–7^. Interestingly, ultrafast endocytosis is compensatory - the membrane area of endocytic vesicles is roughly equal to the total surface area of synaptic vesicles exocytosed^5,6^, suggesting a tight physical coordination between exocytosis and endocytosis.

How are exocytosis and endocytosis coupled at synapses? A previous theoretical study proposes that this coupling is due to a reduction in membrane tension after exocytosis which triggers ultrafast endocytosis^10^. However, membrane tension in neurons seems to propagate at ~20 µm/s^11^, and therefore, is expected to equilibrate at the active zone within 10 ms of exocytosis, well before endocytic pit formation. Therefore, even if membrane tension alters upon exocytosis, this model cannot account for the kinetics and spatial precision of ultrafast endocytosis. Here, we combine experiments and theoretical modeling to address the physical principles of ultrafast endocytosis initiation.

## Results

### Actin filaments are enriched at the periphery of the active zone

Filamentous actin (F-actin) is essential for the initiation of ultrafast endocytosis^5^. When neurons are treated with Latrunculin A, which sequesters monomeric actin (G-actin) and perturbs F-actin formation^12,13^, ultrafast endocytosis fails completely, with no induction of membrane pit formation^5^. However, it is not clear why the actin cytoskeleton is so indispensable.

To probe the role of F-actin in ultrafast endocytosis, we characterized its sub-synaptic localization using 2D stimulated emission depletion (STED) microscopy^14^ and 3D gated-STED (gSTED) microscopy^15,16^. We recently discovered that a splice variant of Dynamin 1, Dyn1xA, forms condensates with Syndapin1^17^ and pre-accumulates at the ‘endocytic zone’ where ultrafast endocytosis takes place, located immediately next to the active zone (within 100 nm of the active zone edge)^18^. Thus, we used Dyn1xA as a proxy for the endocytic zone. We expressed the GFP-tagged calponin-homology domain of F-actin binding protein Utrophin (EGFP-UtrCH)^19^ in cultured mouse hippocampal neurons and visualized its localization relative to the active zone (Bassoon), reserve pool vesicle cluster (Synapsin), and endocytic zone (Dyn1xA^18^). Although UtrCH may not visualize all the F-actin pools uniformly^20^, it has been used previously to probe F-actin pools in axons with higher signal-to-noise than LifeAct^21^. In addition, with Phalloidin staining, presynaptic and postsynaptic actin pools cannot be readily distinguished^22^. Thus, UtrCH was used in this study.

**Fig. 1:**
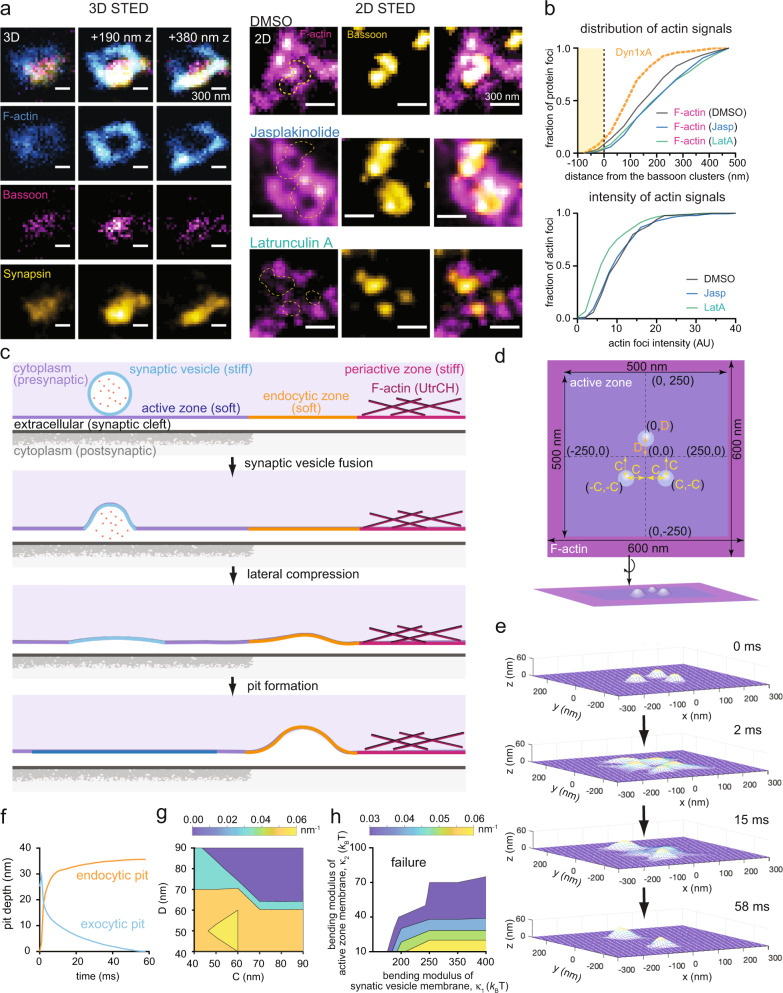
The lateral membrane compression model for ultrafast endocytosis. **a** (left) An example of actin ring distribution in presynapses by 3D STED. The first row shows an overlay of F-actin, Bassoon and Synapsin signals in z-slices separated by 190 nm. Each following row isolates a single fluorescence channel. F-actin is labeled by neuronal expression of EGFP-UtrCH, which is stained by a GFP-antibody and its secondary antibody conjugated to Atto488, Bassoon is labeled by an anti-Bassoon antibody and a secondary antibody conjugated to Atto647N, and Synapsin is labeled by a secondary antibody conjugated to Alexa594. (right) Example 2D STED micrographs showing the localization of filamentous actin (F-actin) relative to the active zone in neurons treated with DMSO (control), Latrunculin A (Lat A), and Jasplakinolide. The active zone is marked by an anti-Bassoon antibody and its secondary antibody conjugated with Alexa594. The dashed circles in the left panels indicate locations of the Bassoon signals. **b** Cumulative plots showing distribution of F-actin signals against the active zone boundary (top) and intensity of F-actin signals (bottom). The active zone boundary was defined by Bassoon signals. See Supplementary Table 2 for detailed statistical analysis. **c** A schematic showing the lateral membrane compression model for ultrafast endocytosis. Only a half of the synapse is depicted for clarity. The lateral membrane pressure exerted by exocytosis is predicted to compress the plasma membrane against the stiff periactive zone membrane and induce pit formation at the interface between actin-free and actin-enriched regions, or at the endocytic zone. The membrane bends towards the cytoplasm likely due to multiple factors like lipid asymmetry and the presence of endocytic proteins inside and postsynaptic membrane outside. Note that the active zone is defined as a region juxtaposed to the postsynaptic density (the gray area along the postsynaptic membrane) where neurotransmitter receptors are localized). **d** Schematics showing the initial conditions of simulations from top-down view (left) and orthogonal view (right). The initial length of active zone (blue) is set at 500 nm. The width of F-actin band (purple) is set at 50 nm. Here, the active zone refers to the actin-free membrane area that includes not only the vesicle fusing area but also the endocytic zone. In contrast, the periactive zone is represented by the F-actin band where actin cortex impinges upon the membrane. The center of the active zone is set as (x, y) = (0, 0). One fusing vesicle (light blue circle) is placed at (0, D), while two other vesicles are placed at (C, -C) and (-C, -C) such that three vesicles would form an isosceles triangle. As the initial condition, we set C = D = 60 nm. **e** Snapshots from simulations, showing the evolution of membrane curvature within the active zone over time. Three fusing vesicles are organized with C = D = 60 nm. At 58 ms, simulations reach the steady state, with 2 endocytic pits forming at the boundary between active zone and actin-enriched region. **f** Plot showing the depth of exocytic pits and endocytic pits as a function of time. **g** (top) Maximal mean curvature of the endocytic membrane pit at maximal pit depth. (bottom) Plot showing the resulting membrane curvature as a function of the spatial arrangement of fusing vesicles. Distances among vesicles are modulated by changing C and D, depicted in **d**. **h** (top) Maximal mean curvature of the endocytic membrane pit at maximal pit depth. (bottom) Plot showing the dependence of successful endocytic pit formation on bending moduli of active zone and periactive zone membranes. The colored areas indicate successful formation of endocytic pits. Source data are provided as a Source Data file.

By 3D gSTED, F-actin is present at presynaptic terminals (Fig. 1a, Supplementary Fig. 1 and 2a for more example micrographs)^22,23^ and typically surrounds the active zone like a ring. However, it is largely excluded from the active zone (Fig. 1a, b), suggesting that the active zone contains little F-actin. F-actin signal peaks further from the edge of the active zone, at 100–200 nm, adjacent to Dyn1xA signals (Fig. 1a, b and Supplementary Fig. 2c). F-actin seems to either distribute like a ring that completely encircles the active zone (64% of synapses), like a partial ring (27% of synapses) that lines 25–75% of the active zone area, or like a patch (9% of synapses) that lines 25% or less of the active zone area (Supplementary Fig. 2a, b). Although few partial rings covered only 25–50% of the active zone area, most partial rings covered 50–75%. Notably, this actin ring is in the plane of the active zone, and when moving out of this plane, a distinct actin structure begins to overlap with the reserve pool cluster, which typically sits directly above or adjacent to the active zone or actin ring (Fig. 1a and Supplementary Fig. 2a). To confirm that EGFP-UtrCH probes F-actin at synapses, we treated neurons with Latrunculin A (10 µM, 1 min) to disrupt its organization or Jasplakinolide (100 nM, 2 min) to stabilize it^24,25^ (Supplementary Fig. 2d, e). After Latrunculin A treatment (Fig. 1a, b), EGFP-UtrCH signals probed by 2D STED decreased in intensity and became less abundant in regions closer to the active zone edge (Fig. 1a, b), suggesting that F-actin is disrupted at presynapses. By contrast, Jasplakinolide treatment shifted actin distribution away from the active zone, likely through enhanced actin polymerization at the periphery, but did not reduce the abundance of preexisting structures (Fig. 1a, b). Together, these results suggest that F-actin surrounds the active zone and endocytic zone like a ring, in the region hereafter referred to as the ‘periactive zone’ (Fig. 1b, c).

### A lateral membrane compression model for exo-endocytic coupling

With this geometry of F-actin at presynapses as a basis, we developed the lateral membrane compression model to explain the precise timing and location of ultrafast endocytosis. This model posits that fused vesicles press laterally onto the active zone membrane, causing the membrane buckling at the interface between the actin-free (active zone) and actin-enriched regions (periactive zone; Fig. 1c). The physical basis of this model hinges on two key assumptions. First, the actin ring at the periactive zone conserves the total membrane area due to the impediment of lipid flow^26–28^. Second, the membrane of fused vesicles is stiffer than the active zone membrane (Fig. 1c, d)^29,30^. This assumption is based on the observations that synaptic vesicle membrane is enriched in components such as cholesterol, sphingomyelin, and transmembrane proteins that increase the bending modulus of the membrane^31–33^. Hereby, synaptic vesicle proteins and lipids are expected to largely remain within the fused vesicle membrane at the millisecond timescale due to their slow intrinsic diffusion ( ~0.1 μm^2^/s or less)^34–36^, high membrane curvature at the neck of fusing vesicles, and the presence of fusion proteins at the pore^37,38^, potentially hindering diffusion.

To translate this qualitative picture into quantitative formulation, we adopted a Helfrich-like energy function to describe the mechanical energy of our membrane system, which includes contributions from bending energy and osmotic pressure (see Methods and Supplementary Table 1). The membrane tension is treated as the Lagrange multiplier that imposes the conservation of the total membrane area. The subsequent dynamics of membrane shape changes are computed by the steep descent of the mechanical energy of the membrane (Supplementary Fig. 3a–d and Supplementary Movies 1, 2). The typical timescale of the dynamics is set by the effective viscous drag coefficient of membrane shape changes (see Methods for details).

We simplified the presynapse to consist of the basic physical parameters we can experimentally access. The model simulations start with fused vesicles that are 40 nm in diameter^5^ and have nearly halfway collapsed into the plasma membrane ( ~26 nm exocytic pit depth). The bending modulus of the synaptic vesicle membrane is set between 100 and 400 *k*_B_T, with the nominal value at 300 *k*_B_T. The linear dimension of the active zone is set between 100 and 700 nm (Fig. 1d), matching the observed parameters in mouse hippocampal synapses (average ~300 nm in diameter)^5,39^. The bending modulus of the active zone membrane is set between 10 and 100 *k*_*B*_T^33,40,41^. The endocytic zone is assumed to have the same bending modulus as the active zone membrane and thus, is included as a part of the active zone (note that no vesicles fuse in this region; Fig. 1c, d). The actin-enriched periactive zone is set at a width of 100 nm with the outer boundary being pinned (Fig. 1c); its associated bending modulus is set between 100 and 400 *k*_*B*_T because cortical actin stiffens the underlying plasma membrane^42–44^. In total, the presynaptic membrane area, which contains the fused vesicles, the active zone and periactive zone, spans 200–800 nm in two dimensions.

Without explicitly describing endocytosis, we used the formation of resulting pits as a proxy for ultrafast endocytosis. We assumed that when the curvature of the resulting membrane pits is sufficiently large ( >0.03/nm), endocytosis would proceed by well-defined curvature-sensing mechanisms of endocytic proteins^45–48^, some of which form condensates just outside the active zone^17,49^. From this perspective, we leveraged our model to dissect the physical mechanisms coupling exocytosis to ultrafast endocytosis at synapses.

As a nominal case, we simulated three synaptic vesicles fusing with the active zone membrane (Fig. 1e), reflecting the average number of vesicle fusions after a single action potential with 4 mM calcium (Ca^2+^) in the external solution^39^. This calcium concentration is used in most of our previous experiments to study ultrafast endocytosis^5,7,49,50^. Our simulations show that as vesicles flatten out, the active zone membrane starts to deform, generating lateral compression against the periactive zone membrane. By ~30 ms, this deformation turns into membrane pits at the interface between the active zone and the periactive zone (Fig. 1e, f; Supplementary Fig. 3a; Supplementary Movies 1, 2). Importantly, the timing and the location of endocytic pit formation quantitatively recapitulates endocytic events observed by ultrastructural analysis^5,6^. The resulting curvature of endocytic pits depends on locations of fused vesicles (Fig. 1g); the further they are apart from each other, the shallower the resulting endocytic pits are. Furthermore, if synaptic vesicle membrane is too soft (below 230 *k*_B_T) or active zone membrane too stiff (above 50 *k*_B_T), endocytic pits do not form, further suggesting that relative stiffness is key to mechanical coupling (Fig. 1h). Importantly, this endocytic pit formation is akin to buckling instability and sensitive to the active zone membrane bending modulus with an abrupt transition occurring at ~110 *k*_B_T, beyond which the formation fails (Supplementary Fig. 3b).

### Membrane area conservation by stable F-actin is necessary for ultrafast endocytosis

**Fig. 2:**
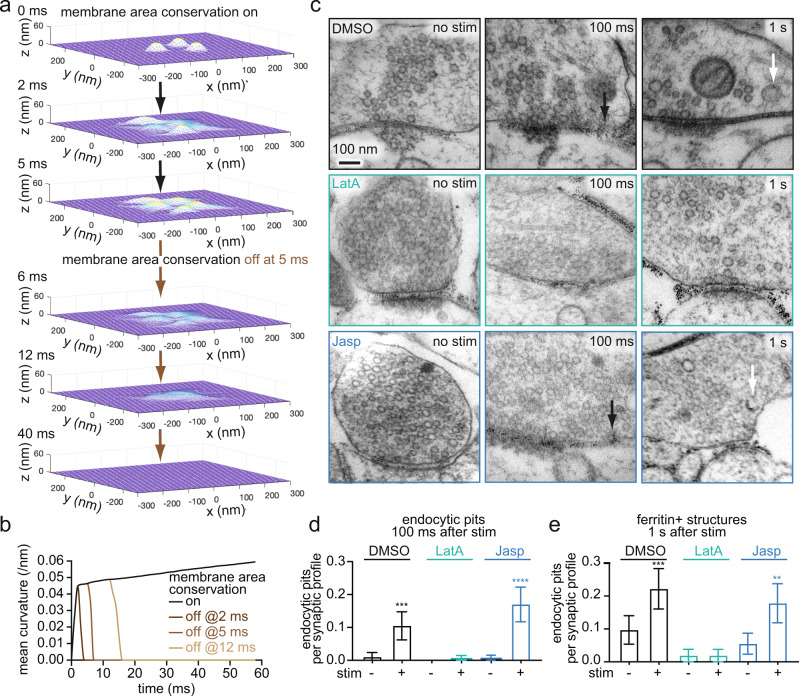
Ultrafast endocytosis is mediated by the membrane area conservation imposed by F-actin. **a** Snapshots from simulations, showing the evolution of membrane curvature within the active zone by fusion of three vesicles when the membrane area conservation is initially on but turned off at 5 ms. Note that the bending moduli of the active zone and periactive zone are made uniform at 20 *k*_B_T when the membrane area conservation is turned off during simulations. **b** Plot showing the temporal evolution of endocytic membrane curvature when the membrane area conservation is turned off at the indicated time points. **c** Electron micrographs showing ChetaTC-expressing wild-type neurons, treated with 0.1% DMSO, 10 µM Latrunculin A (LatA), and 100 nM Jasplakinolide. The left panel show unstimulated conditions, while the right panel show 100 ms after single stimulus (10 ms light pulse, 37 °C, 4 mM external Ca^2+^). Black arrow: endocytic pit. **d** Plot showing the number of endocytic pits at 100 ms after stimulation in unstimulated (-) and stimulated (+) synapses, treated with indicated drugs. Kruskal-Wallis test, with Dunn’s multiple comparisons test. ***p < 0.001. ****p < 0.0001. *p*-values are only shown for direct comparison between unstimulated and stimulated neurons treated with the same drug. See Supplementary Table 2 for the detailed numbers for each sample. **e** Plot showing the number of ferritin-positive endocytic vesicles and endosomes at 1 s after stimulation in unstimulated (-) and stimulated (+) synapses, treated with indicated drugs. Mean and 95% confidential interval are shown. Kruskal-Wallis test, with Dunn’s multiple comparisons test. ***p < 0.001. **p < 0.01. *p*-values are only shown for direct comparison between unstimulated and stimulated neurons treated with the same drug. See Supplementary Table 2 for the detailed numbers for each sample. Source data are provided as a Source Data file.

The membrane compression model predicts that the role of actin filaments in ultrafast endocytosis is passive - they are only needed to conserve membrane area, and thus stable actin polymers are sufficient. Indeed, our simulations show that the persistence of pit formation fails as soon as the membrane area conservation is removed (e.g., at 2 ms, 5 ms, or 12 ms in Fig. 2a, b and Supplementary Movie 3). Thus, the actin ring surrounding the active zone is likely essential for ultrafast endocytosis^5^.

To experimentally test the importance of the membrane area conservation by F-actin, we reduced the total pool of F-actin with Latrunculin A (10 µM, 1 min) (Supplementary Fig. 2d) and performed flash-and-freeze experiments (4 mM Ca^2+^, 37 °C). As in previous experiments^5^, we applied a single 10 ms light pulse (488 nm) to neurons expressing a variant of Channelrhodopsin (ChetaTC)^51^ and froze them at 100 ms or 1 s after stimulation, with unstimulated samples serving as controls. Ferritin particles (2 mg/ml, 5 min) are applied exogenously before freezing to track recently endocytosed structures^5^. Samples were then processed for electron microscopy (see Methods for details). Consistent with our previous work^5^, treatment with DMSO (control) did not affect ultrafast endocytosis, while Latrunculin A treatment completely blocked it (Fig. 2c, d; more micrographs in Supplementary Fig. 4). In Latrunculin A-treated neurons, no endocytic pits formed, and consequently, ferritin-positive endocytic structures did not accumulate in synaptic terminals by 1 s (Fig. 2c, e). These data suggest that the presence, rather than the dynamics, of actin cortex underlies ultrafast endocytosis.

Endocytosis typically requires force generation by active actin polymerization at the maturing endocytic pit^52–57^. The effect of Latrunculin A could be due to the loss of such actin dynamics. By contrast, in our lateral membrane compression model only an intact cytoskeleton is required. To differentiate between these possibilities, we performed the flash-and-freeze experiments in neurons treated with Jasplakinolide (100 nM, 2 min), which stabilizes F-actin and suppresses F-actin turnover^24^ (Supplementary Fig. 2d). Typically, endocytosis that requires active actin polymerization is blocked by both treatments^58–60^. However, ultrafast endocytosis still occurred in neurons treated with Jasplakinolide (Fig. 2c, d). Ferritin particles were internalized into endocytic vesicles and endosomes by 1 s (Fig. 2c, e), suggesting that the initiation and kinetics of ultrafast endocytosis are unaffected by Jasplakinolide. These data suggest that a stable actin cortex, but not dynamic actin polymerization, is necessary for ultrafast endocytosis.

### Two or more vesicles must fuse to trigger ultrafast endocytosis

**Fig. 3:**
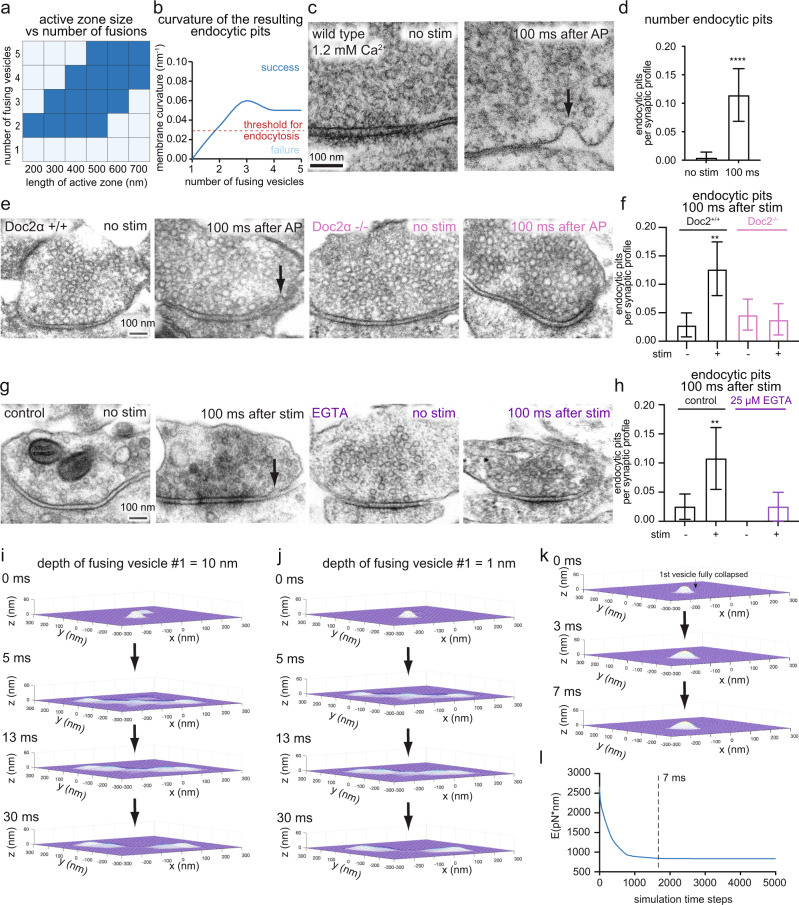
Successful exo-endocytosis coupling requires two or more vesicles to fuse simultaneously or sequentially. **a** Phase diagram showing the relationship between the number fusing vesicles and dimension of the active zone for successful formation of endocytic pits (dark blue). **b** Plot showing curvature of the resulting endocytic pits as a function of the number of fusing vesicles. The red dotted line indicated the threshold for endocytic proteins to recognize the curvature for further maturation of pits into vesicles. **c** Example micrographs showing wild-type synapses unstimulated (left) or stimulated with electric field for 1 ms and frozen 100 ms later (right). The external calcium concentration is 1.2 mM. Black arrow: endocytic pit. **d** Number of endocytic pits at 100 ms after stimulation. Mean and 95% confidential interval are shown. ****p < 0.0001. Two-sided Mann-Whitney U test. See Supplementary Table 2 for detailed statistical analysis. **e** Example micrographs showing wild-type and Doc2α knockout synapses unstimulated or stimulated with electric field for 1 ms and frozen 100 ms later. Black arrow: endocytic pit. **f** Number of endocytic pits at 100 ms after stimulation. **p < 0.01. Error bars are SEM. *p*-values are only shown for direct comparison between unstimulated and stimulated neurons of the same genotype. Kruskal-Wallis test, with Dunn’s multiple comparisons test. See Supplementary Table 2 for the detailed numbers for each sample. **g** Example micrographs showing DMSO- or EGTA-AM-treated synapses unstimulated or stimulated with electric field for 1 ms and frozen 100 ms later. Black arrow: endocytic pit. **h** Number of endocytic pits at 100 ms after stimulation. **p < 0.01. Error bars are SEM. *p*-values are only shown for direct comparison between unstimulated and stimulated neurons of the same genotype. Kruskal-Wallis test, with Dunn’s multiple comparisons test. See Supplementary Table 2 for the detailed numbers for each sample. **i**, **j** Snapshots from simulations, showing the evolution of membrane curvature within the active zone when the second vesicle is fused when the depth of first vesicle is at 10 nm (**i**) and 1 nm (**j**). **k** Snapshots from simulations, showing the evolution of membrane curvature within the active zone when the second vesicle is fused much later, after the first vesicle completely flattens out by relaxing the membrane area conservation. The membrane area conservation is resumed for the second fused vesicle. **l** Simulation result showing the mechanical energy evolution of the system over simulation timesteps, corresponding to (**i**). The energy is equilibrated within ~7 ms. Source data are provided as a Source Data file.

The lateral membrane compression model predicts that for exocytosis-induced lateral compression to trigger endocytosis, there must be a proper scaling between the number of fused vesicles and the dimensions of presynaptic terminals (Fig. 3a, b). If too many vesicles attempt to fuse in a small presynaptic terminal, flattening out synaptic vesicle membrane would incur a high bending energy penalty. Consequently, the synaptic vesicles would not fully collapse and endocytosis would not initiate. On the other hand, if too few vesicles fuse in a large presynaptic terminal, the lateral membrane compression would be insufficient to induce significant curvature in the endocytic zone.

We simulated a varying number of fused vesicles while holding presynaptic terminal size constant and tested whether endocytic pits form. When we simulated two fused vesicles within typically sized active zones (200–500 nm), endocytic pits formed normally, with a curvature of 0.03 nm^−1^ (Fig. 3a, b). By contrast, when only one synaptic vesicle fuses, it cannot completely flatten out unless the membrane area conservation is relaxed (Supplementary Fig. 5a). The lateral membrane pressure from a single vesicle is insufficient to invoke the endocytic pit formation; so instead, the mechanical energy of the membrane reaches a steady-state with incomplete vesicle flattening (Supplementary Fig. 5a; curvature = 0.009 nm^−1^ at 7 ms). On the other hand, when 4 vesicles fuse, endocytic pits form only if the size of the active zone is larger than 400 nm. Similarly, when 5 vesicles fuse, the active zone size must be larger than 500 nm to accommodate extra membrane to ensure successful pit formation (Fig. 3a). Thus, the number of fusion events must be correlated with the size of presynaptic terminals^61^ for successful exo-endocytosis coupling.

**Fig. 4:**
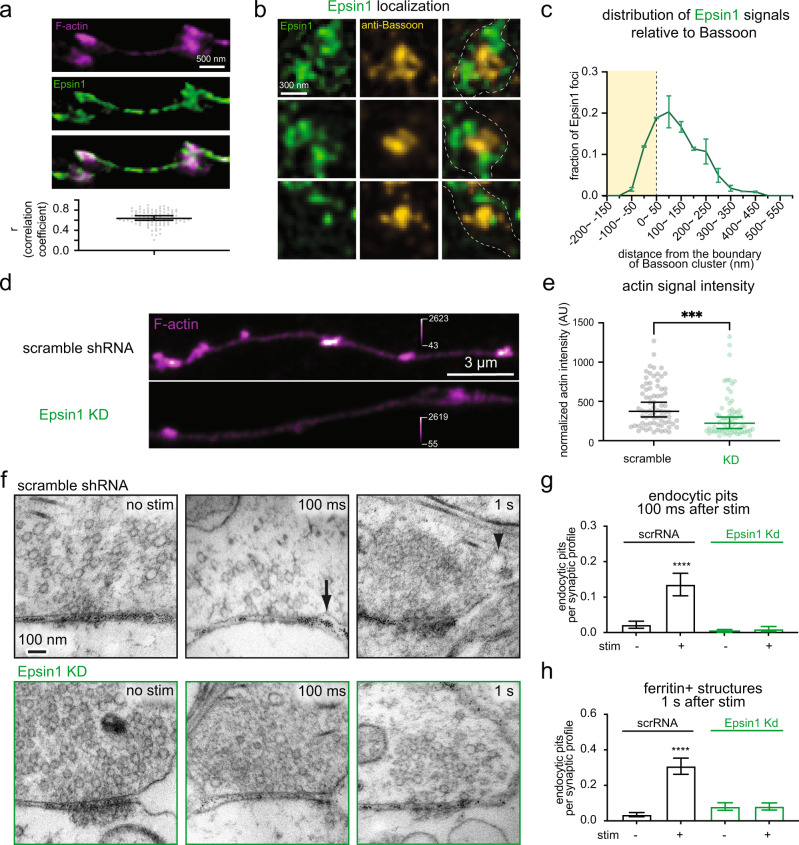
Epsin1 organizes F-actin at presynaptic terminals and is required for ultrafast endocytosis. **a** (top) Example airyscan micrographs showing the localization of Epsin1 relative to F-actin in wild-type neurons. Epsin1 stained with Epsin1-antibody and its secondary antibody conjugated with Atto647N. F-actin is labeled by neuronal expression of EGFP-UtrCH, which is stained by a GFP-antibody and its secondary antibody conjugated to Atto488. (bottom) Plot showing the Pearson’s correlation coefficient. Each dot = synapse. *n* = 94 synapses. *N* = 3 cultures. **b** Example 2D STED micrographs showing the localization of Epsin 1 relative to the active zone in wild-type neurons. Active zone is marked by anti-Bassoon antibody and its secondary antibody conjugated with Alexa594. Epsin1 stained with Epsin1-antibody and its secondary antibody conjugated with Atto647N. **c** The distribution of Epsin1 signals against the active zone boundary. The active zone boundary was defined by Bassoon signals. See Methods for the analysis method. Error bars are SEM. *N* = 2 cultures, *n* = 30–60 per culture. **d** Example confocal fluorescence micrographs showing F-actin signals in neurons expressing scramble shRNA and Epsin1 knock-down (KD) shRNA. F-actin is stained by expressing EGFP-UtrCH. **e** The normalized intensity of F-actin signals from neurons expressing scramble (scr) shRNA, or Epsin1 shRNA, measured by Airyscan. Signals are normalized to the fluorescence signals in axons. Kruskal-Wallis test, with Dunn’s multiple comparisons test. ***p < 0.001. See Supplementary Table 2 for the detailed numbers for each sample. **f** Example electron micrographs showing wild-type and Epsin1 KD synapses unstimulated or stimulated with a single electrical pulse (1 ms) and frozen 100 ms or 1 s later. Black arrow: endocytic pit. Black arrowhead: ferritin-positive endosomes. **g**, **h** Number of endocytic pits at 100 ms after stimulation (**g**) or ferritin-positive structures at 1 s after stimulation (**h**) in neurons expressing scramble shRNA (scrRNA) or Epsin1 shRNA. Mean and 95% confidential interval are shown. Kruskal-Wallis test, with Dunn’s multiple comparisons test. ****p < 0.0001. p-values are only shown for direct comparison between unstimulated and stimulated neurons treated with the same drug. See Supplementary Table 2 for the detailed numbers for each sample. Source data are provided as a Source Data file.

These simulations predict that ultrafast exo-endocytosis coupling requires at least two synaptic vesicles to fuse. To test this prediction, we performed time-resolved electron microscopy analysis of mouse hippocampal neurons using zap-and-freeze^39^. With this approach, we can deliver an electrical pulse of 1 ms to trigger a single action potential and freeze the stimulated neurons at defined time points after stimulation. Using zap-and-freeze, we previously found that fusion of multiple vesicles at a single active zone per action potential (multivesicular release) is common in cultured mouse hippocampal synapses^62,63^. However, with a physiological extracellular calcium concentration ( ~1 mM)^64^, only 1–2 vesicles fuse on average in synapses that respond to a single action potential^39,62^. At this calcium concentration, 62% of responding synapses have only one vesicle fusion immediately after stimulation^39^. In contrast, at 4 mM Ca^2+^, which was used in all other experiments described here and in our previous studies, 39% of responding synapses have only one vesicle fusion. Based on our model, the frequency of ultrafast endocytosis would be expected to differ between synapses under these two calcium concentrations. However, ultrafast endocytosis occurred at a similar frequency in 1.2 mM extracellular calcium (Fig. 3c, d, Supplementary Fig. 6a; Fig. 3e, f: Doc2α +/+) as in 4 mM calcium (Fig. 3g, h: DMSO control and Fig. 4f, g). Thus, there is disparity between theoretical and experimental results.

One potential contributor to this disparity may be asynchronous fusion of synaptic vesicles. Synaptic vesicles can fuse synchronously within a few milliseconds of an action potential and asynchronously thereafter over tens and hundreds of miliseconds^65^. Based on previous studies with zap-and-freeze, patch-clamp electrophysiology measurements, and glutamate imaging, ~35% of the total release that occurs after a single action potential is contributed by this asynchronous component in mouse hippocampal excitatory synapses^62,66^. To test whether asynchronous release contributes to ultrafast endocytosis, we performed zap-and-freeze experiments in a mutant lacking Doc2α, a calcium sensor that mediates asynchronous release after a single stimulus^66,67^, or in wild-type neurons treated with either DMSO (control) or EGTA-AM, which also blocks asynchronous release. Ultrafast endocytosis failed to initiate after a single action potential in Doc2α knockout (Fig. 3e, f; Supplementary Fig. 6a, b) or in EGTA-treated neurons (Fig. 3g, h), suggesting that asynchronous release helps induce ultrafast endocytosis.

Considering this observation, we next adapted the model to fuse two vesicles sequentially instead of simultaneously. The model starts with two fused vesicles: one nearly flattened out (the depth of pits: 1–10 nm) and another just starting to collapse (Fig. 3i, j, Supplementary Movies 4, 5). This simulation suggests that fusion of an additional vesicle leads to the formation of membrane pits if the second vesicle fuses before the first completely flattens out ( ~10 ms after an action potential^39^) (Fig. 3i, j). By contrast, when the second vesicle fuses much later, each event acts independently, as in single vesicle fusion (Fig. 3k, Supplementary Movie 6). In this case, each fused vesicle cannot completely flatten out by itself, unless the membrane area conservation is relaxed. Consequently, no endocytic pits would form, regardless of how lipids and proteins diffuse out of the first vesicle’s membrane (Fig. 3i, j). Therefore, our model suggests that asynchronous fusion likely contributes to ultrafast endocytosis under physiological, low release probability conditions. Given that synapses do not always fuse two vesicles either simultaneously or sequentially before the first completely flattens out, some exocytic events may not trigger successful ultrafast endocytic events. When ultrafast endocytosis is blocked, a slow clathrin-mediated endocytic pathway can kick in^7^ - this is consistent with observations from live-cell imaging of endocytosis^68–70^.

### Epsin1 localizes F-actin to the periactive zone

F-actin ring organization is essential to the mechanical coupling of exocytosis and endocytosis at synapses by conserving the membrane area within the active zone and endocytic zone. What actin regulators dictate F-actin organization at synapses? Epsin1 has been implicated in endocytosis at synapses^71^ and can interact with both the plasma membrane and F-actin through its N-terminal ENTH domain and C-terminal tail, respectively (Supplementary Fig. 7a)^72^. If Epsin1 is important for F-actin organization, it should localize to the periphery of the active zone. To test this prediction, we determined its localization at synapses using fluorescence microscopy (Fig. 4a–c)^49^. Like F-actin (Fig. 1a, b), Epsin1 is localized around the endocytic zone and the periactive zone (Fig. 4b, c). Furthermore, Epsin1 overlaps substantially with F-actin at synapses (Fig. 4a: Pearson’s Correlation coefficient = 0.64). To test whether Epsin1 is required for the normal enrichment of F-actin at this site, we generated an shRNA against Epsin1. Western blot analysis in mouse hippocampal cultures showed that Epsin 1 expression was reduced by 92 ± 8 % at 14 days in vitro (DIV) when neurons were infected with lentivirus carrying shRNA on DIV7 (Supplementary Fig. 7b). Like in neurons treated with Latrunculin A (Fig. 1a, b), Epsin1 knock-down (KD) neurons displayed a 41% reduction in the F-actin level at presynaptic terminals (Fig. 4d, e). These data suggest that Epsin1 localizes F-actin to presynaptic boutons.

Our model predicts that ultrafast endocytosis would fail when actin organization is disrupted, *e.g*., by Latrunculin A treatment (Fig. 2c, d). To test if organization of F-actin by Epsin1 is similarly required, we performed zap-and-freeze experiments in Epsin1 KD neurons (4 mM Ca^2+^, 37 °C). In Epsin1 KD neurons, endocytic pits failed to form within 100 ms (Fig. 4f, g, and Supplementary Fig. 7c), and no ferritin-positive endocytic vesicles and endosomes were observed at 1 s after stimulation (Fig. 4f, h). Together, these results suggest that Epsin1 organizes F-actin at the presynaptic boutons for ultrafast endocytosis.

## Discussion

We identified two essential mechanical factors for ultrafast exo-endocytosis coupling at neuronal synapses. First, flattening of fused vesicles causes lateral pressure against the actin-enriched periactive zone. Second, the actin ring enforces conservation of the total membrane area of the active zone and endocytic zone following exocytosis. This area conservation rectifies the compression, resulting in the ultrafast formation of endocytic membrane pits at the edge of the active zone. Thereafter, endocytic proteins such as Synaptojanin 1, Endophilin A, Syndapin 1, and Dynamin 1 can further curve the membrane and generate vesicles^47–50^.

### Membrane stiffness and tension

The model assumes collapsing synaptic vesicles to be stiffer than the active zone membrane due to high protein density and enrichment of cholesterol and sphingomyelin lipids^73^. Further, the effective stiffness of fused vesicles is likely maintained on a time scale of milliseconds by an inhibition of protein and lipid diffusion due to highly curved membrane at the neck of fused vesicles. On a longer timescale, the synaptic vesicle components will likely mix with the active zone and hence affect the active zone stiffness. However, even if this effect exists, it will be largely diluted because the active zone area is > 100 times larger than synaptic vesicle area. Furthermore, synaptic vesicle components are constantly retrieved by endocytosis. Therefore, such an increase in the active zone stiffness is expected to be marginal and transient. Although a reasonable assumption, measuring the membrane stiffness of synaptic vesicles, presynaptic active zones, and periactive zones is intractable by current technologies. Testing this assumption will be a priority for our future work.

Moreover, the membrane tension of fused vesicles may differ from the active zone membrane and thereby influence exo-endocytosis coupling. For instance, one may assume that synaptic vesicle fusion increases local membrane area, resulting in a spatial gradient of membrane tension; low at the site of fusion and high at the edge of active zone. Indeed, a substantial spatial gradient of membrane tension is thought to persist due to attachment of cytoskeleton to the membrane^28^. However, this spatial gradient is expected to relax within ~1–10 ms of vesicle fusion, since neither the active zone nor collapsing vesicles attach to cytoskeleton. This estimation suggests that the difference in the membrane tension, if it exists, may equilibrate well before the initiation of endocytic membrane pit formation ( ~30–50 ms). Hence, membrane tension cannot account for the location and the timing of ultrafast endocytosis. Consistent with this notion, recent experiments showed that membrane tension equilibrates rapidly at presynaptic terminals^11^. Nonetheless, membrane tension may influence the global conservation of membrane area, thus contributing to ultrafast exo-endocytic coupling.

Our membrane compression model strongly implies the importance of temperature in determining what mode of synaptic vesicle endocytosis will occur. In our previous study, we demonstrated that ultrafast endocytosis fails when neurons are incubated at room temperature for 5 min prior to experiments – this failure is compensated by clathrin-mediated endocytosis^7^. Based on the reliance of ultrafast endocytosis on F-actin^7^ and the high sensitivity of actin polymerization to temperature change^74–76^, we attribute this result to a slowed actin turnover at the room temperature^7^. However, such a drastic change in the temperature would also lower the membrane fluidity, in effect stiffening the plasma membrane globally. Since the ultrafast formation of endocytic pits critically depends on the active zone membrane being softer than the vesicle membrane (Fig. 1h and Supplementary Fig. 3b), ultrafast endocytosis may fail under such conditions. Still, excess vesicle proteins are likely recognized by clathrin-adaptors and then internalized via clathrin scaffolds. Thus, besides the kinetics of proteins involved in endocytosis, changes in the membrane biophysics must be considered when experiments are performed at non-physiological temperatures.

### Asynchronous release and its importance for endocytosis at synapses

In the lateral membrane compression model, the number of fused vesicles and the resulting magnitude of the compressive force are important for triggering ultrafast endocytosis. In the various sizes of active zones we simulated, two fused vesicles are the minimal requirement. However, two vesicles do not need to fuse simultaneously as one can fuse after the first one has begun to flatten. In excitatory mouse hippocampal neurons, the calcium sensor Doc2α drives the majority of asynchronous release after a single action potential^66,67^. Strikingly, in the absence of this sensor, ultrafast endocytosis fails completely (Fig. 3e, f), suggesting that Doc2α-mediated exocytosis is a key contributor to the initiation of membrane buckling at the endocytic zone. When ultrafast endocytosis is blocked, clathrin-mediated endocytosis takes its place^7^. Thus, in synapses with fewer fusions, endocytosis still occurs, but is expected to be slow. Consistent with this notion, several previous studies suggest that endocytosis becomes slower when neurons are treated with the high-affinity calcium chelator EGTA, which abolishes asynchronous release^77–81^. It is possible that calcium directly accelerates endocytosis, as previously suggested^77–81^. However, given that calcium dynamics and calcium sensors regulate exocytosis, the role of calcium in endocytosis may need to be reassessed. Indeed, in the absence of calcium, presynaptic endocytic pits reminiscent of ultrafast endocytosis form when exocytosis is induced with a hyperosmotic shock^10^.

### F-actin organization at presynaptic terminals

The organization of F-actin is essential to the lateral membrane compression model by enforcing the membrane area conservation and restricting lipid flow. We found that F-actin is arranged in a ring-like structure surrounding the active zone. These results differ from those of a recent study using single-molecule localization microscopy of presynaptic terminals formed on polystyrene microbeads. In that system, three pools of F-actin were apparent: actin rails between the active zone and vesicles, actin corrals around the terminal^22^, and in contrast to our results, an actin mesh at the active zone. The presence of F-actin in such artificially-formed active zones may reflect cytoskeletal response due to changes in cortical tension, since a bead is much stiffer than a postsynaptic terminal^82^. F-actin was rarely observed within the active zone in our experiments. Thus, active zones likely contain little F-actin. This spatial arrangement of F-actin at mouse hippocampal boutons is similar to presynaptic terminals of lamprey recticulospinal cord^83^ and *Drosophila* neuromuscular junctions^84^.

### The role of actin in endocytosis

The role of actin in presynaptic endocytosis has been elusive. In yeast, active force generation by actin polymerization has been long established as the mechanism by which actin supports endocytosis^85,86^. Due to the high turgor pressure impinging upon yeast plasma membranes, this force generation is essential to invaginate the endocytic membrane. However, in the case of much softer animal cell membranes, the role of actin polymerization in endocytosis is controversial^55^. Nevertheless, genetic ablation of actin in mammalian neurons strongly perturbs endocytosis;^87^ like in yeast, active actin polymerization may be important for endocytosis at synapses. Our work suggests that 1) actin is organized in a ring-like structure surrounding the active zone (Fig. 1a), with the endocytic zone also largely free of F-actin, and 2) intact F-actin, but not its dynamic polymerization, is essential for the initiation of ultrafast endocytosis (Fig. 2c, d). In the lateral membrane compression model, stable F-actin is proposed to conserve the membrane area. However, this does not exclude the possibility that actin dynamics are necessary for ultrafast endocytosis. In fact, ultrafast endocytosis fails within 30–60 s of Latrunculin A treatment, indicating that the turnover of F-actin is rapid. Additionally, recent work suggests that the actin nucleator formin is required for synaptic vesicle endocytosis at small hippocampal boutons^8^. Therefore, dynamic actin polymerization may be important for maintaining the cortical F-actin organization in the periactive zone.

### Mechanisms of F-actin organization at synapses

Epsin1 seems to play a key role in actin organization at synapses. Specifically, knocking down Epsin1 in mouse hippocampal neurons results in 1) a reduced amount of F-actin around the periactive zone and 2) failure of ultrafast endocytosis. We interpret these results to indicate that Epsin1 is the organizer of cortical F-actin at synapses for ultrafast endocytosis. However, it is not clear whether Epsin1 is the sole organizer of F-actin at synapses, since it interacts with other endocytic proteins such as Eps15 and Hip1R^72,88^, both of which interact either directly or indirectly with membranes and actin. Thus, Epsin1-interacting proteins may also have roles in actin organization. In addition, the first alpha helix (α0) of the N-terminal ENTH domain can insert into the membrane and induce curvature formation (Fig. 4a)^89,90^ – this function of Epsin1 is thought to be important for endocytosis. Thus, Epsin1 may have a more active role in ultrafast endocytosis beyond organizing F-actin. Nonetheless, our results here suggest that the first step in ultrafast endocytosis at synapses does not directly involve proteins, but rather is initiated by an excess of ‘stiff’ vesicle membrane in an active zone.

### Ultrafast mechanics in biological functions

Conceptually, our work provides an example of how mechanics can be exploited to speed up biological functions. With the actin ring encircling and conserving the membrane area, it enables the release of mechanical energy stored in stiff synaptic vesicles. Like a loaded spring, fusion of these stiff vesicles causes rapid membrane buckling and endocytosis at the edge of the softer active zone. In a broader biological context, similar mechanical systems have been demonstrated at the molecular level: For example, the F1 ATPase (the smallest rotary motor) uses its beta-sheet as a spring to store energy from ATP, and then rapidly releases it to create the high-speed rotary torque ( ~100 Hz)^91^. Likewise, organisms exploit the same strategy to catch their prey. For instance, a Venus flytrap leaf preloads its trap and then snaps it at a 100 ms timescale via a buckling instability^92^. Our finding adds to this general picture, but at the cellular level. Despite the overwhelming role of chemical signaling in the nervous system, underpinning the efficacy of this cell-cell communication is a highly organized presynaptic mechanical landscape that is structured to react rapidly to local mechanical changes.

## Methods

### Animal use

All the animal work was performed according to the National Institutes of Health guidelines for animal research with approval from the Animal Care and Use Committees at the Johns Hopkins University School of Medicine. Mice are maintained in accordance with the Johns Hopkins Animal Care and Use Committee regulations. These include ad libitum access to food and water, sterile environment with enrichment items, weekly cage changes, temperature control at 22 °C, and a 12 h light/ 12 h dark cycle. For Doc2α knockout experiments, C57BL/6-*DOC2a*^*em1Smoc*^ ^93^ were maintained as heterozygotes, and neurons were cultured from homozygous null P0 pups, with homozygous WT littermates used as controls. For all other experiments, neurons were cultured from E18 embryos from C57BL/6 J mice.

### Primary neuron culture

Primary hippocampal neurons were isolated from either E18 embryos or P0 pups of both genders. The brains were harvested from animals and hippocampi were dissected under a binocular microscope. Dissected hippocampi were collected in ice-cold dissecting media (1 x HBSS, 1 mM sodium pyruvate, 10 mM HEPES, pH7.2–7.5, 30 mM glucose, 1% penicillin-streptomycin) and later digested with papain (0.5 mg/ml) and DNase (0.01%) for 25 min at 37 °C. Cells were dissociated by trituration using fire-polished Pasteur pipettes.

For high pressure freezing experiments neurons were plated onto 6 mm sapphire disks (Technotrade Inc) coated with poly-D-lysine (1 mg/ml) and collagen (0.6 mg/ml) with an astrocyte feeder layer on it^94^. Cortices were harvested from E18/P0 animals and astrocyte was isolated with a treatment of trypsin (0.05%) for 20 min at 37 °C, followed by trituration. Astrocytes were seeded in T-75 flasks containing DMEM supplemented with 10% FBS and 0.2% penicillin-streptomycin. After 2 weeks, astrocytes (50 K/well) were plated on sapphire disks. After 1 week in culture, astrocytes were incubated with 5-Fluoro-2′-deoxyuridine (81 µM) and uridine (204 µM) for at least 2 h to stop mitosis. Prior to addition of hippocampal neurons, medium was changed to Neurobasal-A (Gibco) supplemented with 2 mM GlutaMax, 2% B27 and 0.2% penicillin-streptomycin. For fluorescence imaging, dissociated hippocampal neurons were seeded onto 18 mm or 25 mm coverslips (Carolina Biologicals) coated with poly-L-lysine (1 mg/ml, Sigma) at a density of 25–40 × 10^3^ cells/cm^2^ in Neurobasal media (Gibco) supplemented with 2 mM GlutaMax, 2% B27, 5% horse serum and 1% penicillin-streptomycin (NM5) at 37 °C in 5% CO_2_. Next day, media were changed to Neurobasal media with 2 mM GlutaMax and 2% B27 (NM0), and neurons were maintained in this medium until use. For biochemical experiments, dissociated hippocampal neurons were seeded on poly-L-lysine (1 mg/ml) coated plates with Neurobasal media supplemented with 2 mM GlutaMax, 2% B27, 5% horse serum and 1% penicillin-streptomycin, at a density of 1 × 10^5^ cells/cm^2^. Next day, the medium was changed to Neurobasal medium containing 2 mM GlutaMax and 2% B27 (NM0), and neurons were maintained in this medium. A half of the media was refreshed every week or as needed. For Doc2a KO and WT littermate cultures, tail clips were obtained from live P0 pups and genotyped as described in ref. ^62^. Brain tissues were harvested from correct genotypes and hippocampal neurons were prepared as described above.

### Plasmids

For knocking down Epsin1, a plasmid (pLKO.1-puro backbone) containing shRNA sequence (CCGGGATGAAGAATATCGTCCACAACTCGAGTTGTGGACGATATTCTTCATCTTTTTG) was purchased from Sigma (Mission shRNA, clone ID TRCN0000111703). For non-targeting scramble control, oligo’s containing sequence (GATCCCTTCGCACCCTACTTCGTGGttcaagagaCCACGAAGTAGGGTGCGAATTTTTGGAAATTAAT) was cloned under U6 promoter. Annealed oligo’s were inserted into BamH1 and PacI sites of modified pFUGW vector using TAKARA solution I. For labeling actin, we acquired a plasmid containing tandem calponin homology (CH) domains from the human actin binding protein utrophin which are C-terminally linked to GFP from addgene (pEGFP-C1 Utr261-EGFP, #58471). We also C-terminally tagged Utr261 with HaloTag under CMV promoter using in-fusion cloning method. To label the endocytic zone, we used a plasmid expressing Dynamin1xA C-terminally tagged to GFP, purchased from addgene (phsDyn1xA-EGFP-N1, #120313). For visualizing Epsin1, we used a plasmid expressing Epsin1-EGFP, obtained from Addgene (pEGFPC1-Epsin1, #22228).

### Lentivirus preparation

Lentivirus containing either Epsin1 shRNA or nontargeting scramble shRNA was prepared as described earlier in ref. ^49^. Briefly, shRNA construct along with two helper DNA constructs (pHR‐CMV8.2 deltaR (Addgene 8454) and pCMV‐VSVG (Addgene 8455)) at a 4:3:2 molar ratio was transfected into HEK293T cells using polyethylene amine. Cell supernatant containing the virus was collected 3 days after transfection and 20-fold concentrated using Amicon Ultra 15 10 K (Millipore) centrifugal filter. Aliquots were flash frozen in liquid nitrogen and stored in −80 °C until use.

### Transient transfection of neurons

For transient expression of proteins, neurons were transfected at DIV (days in vitro) 14–16 by Lipofectamine 2000 (Invitrogen) according to the manufacturer’s manual. Prior to transfection, half of the media from each well was taken out and mixed with fresh NM0 (see above) that was left to warm to 37 °C and equilibrate with CO_2_ in an incubator (recovery media). The rest of the media was aspirated and replaced with fresh NM0 for transfection. Plasmids were diluted in NeuroBasal Plus (Gibco) media so that 0.5-1 µg of DNA would be added to each well. Prior to addition, DNA was mixed with a solution containing Lipofectamine 2000 such that there was a 1:1 ratio of µg DNA to µL Lipofectamine. This mixture was added to each well and incubated for 4 h. Afterwards, the transfection media was removed and replaced with recovery media previously prepared. After 16-20 hours, neurons were either used for pharmacological treatment or fixed for immunofluorescence.

### Pharmacology

For Actin perturbation experiments, transfected cells were treated immediately prior to fixation. Latrunculin A and Jasplakinolide stock solutions were prepared in DMSO as 10 mM and 100 µM, respectively. Stock drug solutions were diluted into the media in wells at a 1:1000 dilution for a final concentration of 10 µM for Latrunculin A and 100 nM for Jasplakinolide. Cells were treated with Latrunculin A for 30 s to 1 min, while cells were treated with Jasplakinolide for 1.5–2 min. Cells treated with these drugs for 30 min also show similar results. Negative control samples were treated with 0.1% DMSO.

### Immunofluorescence

For immunofluorescence experiments were performed with DIV 14–16 hippocampal neurons. Culture media was removed from the wells and fixed with a prewarmed 1X PBS containing 4% paraformaldehyde and 4% sucrose in for 20 min at room temperature. Following fixation, cells were washed three times with 1x PBS. Next, cells were permeabilized by 0.2% Triton X-100 diluted in 1X PBS for 8 min. After three washes with 1X PBS, cells were blocked by 1% BSA in 1X PBS for 1 h. Then, the coverslips containing neurons were transferred to a humidified chamber and placed face down on a drop of primary antibody solution. Primary antibodies were diluted 1:500 in a 1% BSA 1X PBS and cells were incubated at 4 ^o^C overnight. UtrCH-EGFP was stained by an anti-GFP rabbit polyclonal antibody (MBL International). Endogenous Bassoon protein was stained by an anti-Bassoon mouse monoclonal (Synaptic Systems) antibody. Endogenous Synapsin protein was stained by an anti-Synapsin polyclonal guinea pig (Synaptic Systems) antibody. Next, coverslips were washed with 1X PBS three times. Secondary antibodies were diluted in 1X PBS containing 1% BSA. For superresolution 2D STED imaging, an anti-rabbit Atto647N (Rockland) secondary antibody was used at 1:120 dilution and an anti-mouse Alexa594 (Invitrogen) secondary antibody was used at 1:1000. For 3D gSTED, a 1:500 dilution was used for Atto488, (Rockland), Alexa594, or Atto647N secondary antibodies. Secondary antibody incubation was performed in a humidified chamber as described previously for 1 h at room temperature. Following three 1X PBS washes, cells were rinsed with Milli-Q water and mounted on a glass slide containing a drop of ProLong Diamond Antifade Mounting media (Thermo Fisher). Mounting Media was allowed to solidify for 24 h at room temperature in the dark before proceeding to STED imaging. For confocal imaging in Airyscan, cells were prepared in a similar way.

### Stimulated emission depletion microscopy (STED)

All 2D STED images were obtained using a home-built two-color STED microscope^95^. A femtosecond laser beam with a repetition rate of 80 MHz from a Ti:Sapphire laser head (Mai Tai HP, Spectra-Physics) is split into two parts: one part is used to produce the excitation beam, which is coupled into a photonic crystal fiber (Newport) for wide-spectrum light generation and is further filtered by a frequency-tunable acoustic optical tunable filter (AA Opto-Electronic) for multi-color excitation. The other part of the laser pulse is temporally stretched to ~300 ps (with two 15 cm-long glass rods and a 100 m long polarization-maintaining single-mode fiber, OZ optics), collimated, expanded, and wave-front modulated with a vortex phase plate (VPP-1, RPC photonics) for hollow STED spot generation to de-excite the fluorophores at the periphery of the excitation focus, thus improving the lateral resolution. The STED beam is set at 765 nm with power of 120 mW at the back focal plane of the objective (NA = 1.4 HCX PL APO 100×, Leica), and the excitation wavelengths are set as 594 nm and 650 nm for imaging Alexa-594 and Atto-647N labeled targets, respectively. The fluorescent photons are detected by two avalanche photodiodes (SPCM-AQR-14-FC, Perkin Elmer). The images are obtained by scanning a piezo-controlled stage (Max311D, Thorlabs) controlled by the Imspector data acquisition program.

3D gSTED images were collected by the Leica TCS SP8 with 3x gSTED and TauSTED commercial microscope. Images were collected in gSTED mode.

### Analysis of 3D gSTED images

30 × 30 pixel (2.25 mm^2^) ROIs were selected for actin distribution analysis. For classification as a ring, partial ring or patch, actin signal was visualized in the plane in which Bassoon signal peaked, and in the planes directly above or below. If more than 75% of the Bassoon area is adjacent to actin signal, the presynaptic actin distribution is classified as a ring. If it is less than 75%, but greater than 25%, it is classified as a partial ring. If it is less than 25%, it is classified as a patch.

### Data analysis of 2D STED images

A custom MATLAB code package was used to analyze actin and endocytic protein distribution relative to the active zone marked by Bassoon in STED images^18^. First, STED images were blurred with a Gaussian filter with radius of 1.2 pixels to reduce the Poisson noise, and then deconvoluted twice using the built-in deconvblind function: the first point spread function (PSF) input is measured from nonspecific antibody signal in the STED images, and the second PSF input is chosen as the returned PSF from the first run of blind deconvolution^96^. Each time, 10 iterations are performed. Presynaptic boutons in each deconvoluted image were selected within 30 × 30-pixel (0.81 mm^2^) ROIs based on the varicosity shape and bassoon signal. The active zone boundary was identified as the contour that represents half of the intensity of each local intensity peak in the Bassoon channel, and the actin or endocytic protein clusters are picked as local maxima. The distances between the protein cluster centers and the active zone boundary are automatically calculated correspondingly. Actin and endocytic protein clusters over crossing the ROIs, and the Bassoon signals outside of the transfected neurons were excluded from the analysis. For each condition, roughly 80-100 boutons (n) were quantified from 3 different cultures (N). The MATLAB scripts are available by request.

### Airyscan imaging and data analysis

For Airyscan imaging, samples were imaged in Zeiss LSM880 (Carl Zeiss) in Airyscan mode. Fluorescence was acquired using a 63x objective lens (NA = 0.55) at 2048 × 2048 pixel resolution with the following settings: pixel Dwell 1.02 ms and pin hole size above the lower limit for Airyscan imaging, as computed by ZEN software. To determine colocalization of Epsin1 and F-actin, neurons were co-transfected with Epsin1-EGFP and UtrCH-HaloTag. 20 hours after transfection neurons were labeled with Halo-JFX646 (100 nM) (Janelia Research Campus) for 30 min at 37 °C inside CO_2_ incubator. Cells were washed 4 times (30 min each) with fresh media to remove unbound dyes. Colocalization between Epsin1 and Actin were determined using Pearson’s correlation coefficient derived from ImageJ using coloc2 plugin. For experiments comparing fluorescence intensities between scramble and shRNA treated cells, staining and microscope settings were remained constant. Utr-EGFP was used to stain actin as described before and GFP signal intensity was used to determine actin level. Presynaptic regions were determined with Bassoon-Alexa594 signals. Axons were distinguished from dendritic processes based on their morphology, thin and lacking spines. Z-sections were taken for each presynaptic bouton along axons. Utr-EGFP intensities, depicting actin level in bouton, was quantitated in ImageJ. Bassoon-Alexa594 signal was used to determine the ROI’s, and all Utr-EGFP signals within ROIs were measured as the total signals at each synapse. Fluorescence intensity was normalized to the average signal within the corresponding cell body of each axon.

### High pressure freezing

75 K Hippocampal neurons cultured on sapphire disks were frozen using high pressure freezer (EM ICE, Leica Microsystems). For actin dynamics perturbation, neurons were treated with Latrunculin A (10 µM for 1 min), Jasplakinolide (100 nM for 2 min) and DMSO as control. For EGTA-AM experiments, EGTA-AM (Fisher) or DMSO was added to the medium to a final concentration of 25 μM and 0.25% DMSO. Cells were incubated for 15 min, washed three times, and left in the media for 15 min prior to high-pressure freezing. Epsin1 and scramble shRNA’s were added to cells on DIV7 and freezing experiments were performed on DIV14. Ferritin (2 mg/ml) was used as a fluid-phase marker and added to the cells for 5 min prior to freezing. Cells were frozen in the physiological saline solution (140 mM NaCl, 2.4 mM KCl, 10 mM HEPES, 10 mM glucose; pH adjusted to 7.3 with NaOH, 300 mOsm) containing NBQX (3 µM, Tocris) and bicuculine (30 µM; Tocris), which were added to block recurrent synaptic activity. CaCl_2_ and MgCl_2_ concentrations were adjusted as needed for experiments (mentioned in results section). Zap-and-freeze experiments were performed as described earlier (Kusick et al. 2020). After freezing, samples were transferred under liquid nitrogen to an automated freeze substitution system at −90 °C (EM AFS2, Leica Microsystems). Using pre-cooled tweezers, samples were quickly transferred to anhydrous acetone at −90 °C. After disassembling the freezing apparatus, sapphire disks with cells were quickly moved to cryovials containing freeze substitution solutions and left inside EM AFS2. For EM experiments described in Figs. 2, 4 freeze substitution was performed in solutions containing 1% glutaraldehyde, 1% osmium tetroxide, and 1% water in anhydrous acetone, which had been stored under liquid nitrogen then moved to the AFS2 immediately before use. The freeze substitution program was as follows: −90 °C for 6–10 h (adjusted so substitution would finish in the morning), 5 °C h^−1^ to −20 °C, 12 h at −20 °C, and 10 °C h^−1^ to 20 °C. For EM experiments using neurons from Doc2a KO and EGTA-AM (Fig. 3) a different freeze-substitution protocol was used that generated more consistent results. In this protocol, after freezing samples were first left in 0.1% tannic acid and 1% glutaraldehyde at −90 °C for ~36 hours, then washed 5 times, once every 30 min, with pre-chilled acetone, and transferred to 2% osmium tetroxide in acetone. Freeze substitution program was as follows: 11 hr at −90 °C, 5 °C h^−1^ to −20 °C, −20 °C for 12 hr, 10 °C h^−1^ to 4 °C, then removed from the freeze substitution chamber and warmed at room temperature for ~15 min before infiltration and embedding. For this latter protocol, all the steps were performed in universal sample containers (Leica Microsystems) and kept covered in aclar film to prevent any evaporation.

### Sample preparation for electron microscopy

Following freeze-substitution, fixatives were washed with anhydrous acetone for five times, 10 min each. 100% epon araldite (epon 6.2 g; araldite 4.4 g; DDSA 12.2 g, and BDMA 0.8 ml) solution was prepared and diluted acetone to get 30%, 70 and 90% solutions. Samples were infiltrated for at least two hours at room temperature sequentially in 30%, 70% epon-araldite. Samples were then transferred to caps of polyethylene BEEM capsules with 90% epon araldite and incubate overnight at 4 °C. Next day, samples were transferred to new caps with fresh 100% epon araldite, changed every 2 h three times, after which samples were cured at 60 °C for 48 h.

After resin was cured, 40 nm sections were cut using an ultramicrotome (EM UC7, Leica microsystems) and collected on single-slot copper grids coated with 0.7% pioloform. The sections were stained with 2.5% uranyl acetate in 75% methanol.

### Electron microscopy imaging and data analysis

Samples were imaged on a Hitachi 7600 TEM equipped with an AMT XR50 camera run on AMT Capture v6 (pixel size = 560 pm), at 80 kV on the 100,000x setting. Samples were blinded before imaging. Synapses were identified by a vesicle-filled presynaptic bouton and a postsynaptic density. Postsynaptic densities are often subtle in our samples, but synaptic clefts were also identifiable by 1) their characteristic width, 2) the apposed membranes following each other closely, and 3) vesicles near the presynaptic active zone. 125–150 micrographs per sample of anything that appeared to be a synapse were taken without close examination. All images were from different synapses.

EM image analysis was performed as previously described in ref. ^39^. All the images from a single experiment were randomized for analysis as a single pool. Only after this randomization were any images excluded from analysis, either because they appeared to not contain a bona fide synapse or the morphology was too poor for reliable annotation. The plasma membrane, the active zone, exocytic and endocytic pits, clathrin coated pits docked synaptic vesicles, and all synaptic vesicles in the bouton were annotated in ImageJ using SynapsEM plugins [https://github.com/shigekiwatanabe/SynapsEM copy archived at swh:1:rev:11a6227cd5951bf5e077cb9b3220553b506eadbe] (Watanabe et al. 2020). To minimize bias and error, and to maintain consistency, all image segmentation, still in the form of randomized files, was thoroughly checked and edited by a second member of the lab. Features were then quantitated using the SynapsEM (Watanabe et al., 2020) family of MATLAB (MathWorks) scripts (https://github.com/shigekiwatanabe/SynapsEM). Example electron micrographs shown were adjusted in brightness and contrast to different degrees (depending on the varying brightness and contrast of the raw images), rotated, and cropped in ImageJ before import into Adobe Illustrator.

### Western blot analysis

To test efficiency of Epsin1 shRNA to knock down endogenous protein, lentivirus containing scramble or shRNA was added to cultured hippocampal neurons on DIV7. Neurons were harvested on DIV14 and lysed by addition of lysis buffer (50 mM Tris pH 8.0 and 1% SDS containing cOmplete Mini Protease Inhibitor (Roche)) and boiling at 95 °C for 5 min. Lysates were centrifuged at 15,000 x g for 15 min at 4 °C and the supernatants were separated in SDS–PAGE and transferred onto Immobilon-FL membranes (Millipore). Following blocking with 5% skim milk in PBS containing 0.05% Tween-20 (PBST) for 30 min, membranes were incubated with Epsin 1 (Thermo Fisher, rabbit) and control β-actin (SYSY, mouse) antibodies diluted to 1:1000 and 1:10000 respectively in 3% BSA/PBST overnight at 4 °C, followed by IRDye secondary antibodies (LiCor, anti-mouse 680, anti-rabbit 800) diluted to 1:10,000 in 3% BSA/PBST for 45 min at room temperature. Signal was detected using LiCor Odyssey Clx and quantification was done by Image Studio Lite from LiCor.

### Statistical analysis

Detailed statistical information is collated in table S2. STED images were acquired from ~2 biological replicates per condition. Each replicate was a dissociated mouse hippocampal culture (N) taken from different mice on different days. For each N, roughly 30 presynaptic bouton regions of interest (ROIs) (n) were imaged from multiple transfected cells. ROIs from each replicate were pooled and quantified as previously described in ref. ^17^. An alpha of 0.05 was set for null hypothesis testing. For actin and dynamin1xA foci distance statistical analysis, pooled distance measurements from each condition were assessed for distribution normality. A full-pairwise Kruskal Wallis test was performed. Afterwards, each condition was compared by Dunn’s multiple comparisons test. Intensity measurements taken from the same datasets were assessed for normality. Following this, each condition was compared by a Kruskal-Wallis test for non-parametric distributions, followed by a Dunn’s multiple comparisons test. Non-STED fluorescence experiments were prepared and quantified in the same manner as STED experiments. However, these datasets only required comparison of only two conditions, and therefore, a Mann-Whitney test was used for nonparametric statistical comparison.

For electron microscopy data, measurements were taken from roughly 100 synaptic profile micrographs (n) per condition. Replicate high-pressure freezing experiments (N) were conducted with cultures taken from different mice on different days. Sample sizes for each replicate were inferred from previous flash-and-freeze experiments as opposed to power analysis. An alpha of 0.05 was set for null hypothesis testing. For count data sets such as these, anormal, nonparametric distributions are assumed and typical. However, means are best to represent central tendency, and these data are binomially distributed. So, an ANOVA test with a Brown-Forsythe correction and Games-Howell post hoc was conducted. In the case of electron microscopy data sets with measurements of 0, Brown-Forsythe correction fails. Therefore, statistical comparison by a Kruskal-Wallis test followed by Dunn’s multiple comparisons was used instead. All statistics were performed and data plotted by GraphPad Prism.

### Model simulation methods

The mechanical energy of the membrane system, *E*, is described by a Helfrich-like energy (Eq. (1))^97^, including the bending energy and that from osmotic pressure.

1 $$E={\mathop{\sum}\limits_{i=1}^{3}}\oiint {{ds}}_{i} \left[2{\kappa }_{i}{(\Omega -{\Omega }_{0})}^{2}\right]+\Pi \cdot V$$

Here, the surface integral sums over 3 sub-domains of contiguous membrane with different bending moduli. The first sub-domain (*i* = 1) is the stiff membrane from post-fusion synaptic vesicles;^31,33^ the associated bending modulus, *κ*_1_, is assumed to range from 100 *k*_B_T to 400 *k*_B_T (see Supplemental Table 1). The second sub-domain (*i* = 2) is the soft membrane that includes the active zone and the surrounding endocytic zone where the endocytosis takes place; the associated bending modulus, *κ*_2_, is assumed to be range from 10 *k*_B_T to 100 *k*_B_T (see Supplemental Table 1)^40,41^. The last sub-domain (*i* = 3) is the actin-enriched periactive zone, whose bending modulus, *κ*_3_, is set to be the same as *κ*_1_. However, it is noted that the essential model results largely remain insensitive to the specific choice of *κ*_3_.

$\Omega=({\Omega }_{1}+{\Omega }_{2})/2$ is the mean curvature, where $\Omega$_1_ and $\Omega$_2_ are the curvatures in the two principal directions along the two-dimensional manifold of membrane surface, respectively. ${\Omega }_{0}$ is the spontaneous curvature of the membrane and assumed to be zero. Since our model does not describe the fusion of the exocytosis and endocytosis pinching off, the topology of the membrane patch remains constant and hence, the contribution of the Gaussian curvature is constant and can be omitted^98^.

Additionally, $\Pi$ is osmatic pressure in the presynaptic neuron that points outward, and *V* is the volume sandwiched between the membrane shape deformation and the flat baseline. This way, when the membrane curve inward, it causes the energy penalty from the osmotic pressure.

Note that both the mean curvature $(\Omega )$ and the volume (*V*) in the membrane energy, *E*, of Eq. (1) are functions of the local membrane height, *h*(x, y). And the spatial-temporal evolution of *h*(x, y) depicts the membrane shape changes driven by the gradient of membrane energy (*i.e*., the functional derivative of *E* upon the variations of *h*(x,y) in Eq. (2)):

2 $$\eta \frac{\partial h}{\partial t}=-\frac{\delta E}{\delta h}$$

That is, the evolution of the local membrane height, *h*(x,y), is determined by the minimization of the Helfrich-like energy by descend gradient method. Importantly, the effective viscous drag coefficient of membrane shape changes, $\eta$, sets the timescale of the dynamics.

To numerically compute the membrane shape changes, we used Surface Evolver^99^, which is a software specialized in minimizing the energy of triangulated surfaces^100,101^. In the model simulation, three constraints were implemented. First, the total membrane area, ${\sum }_{i=1}^{3}\oiint {{ds}}_{i}$, was conserved; the membrane tension is treated as the Lagrange multiplier while minimizing the energy in Eq. (2) Notably, the model can modulate this conservation by modulating its duration in the numerical simulation. Second, the outer boundary of the actin-enriched periactive zone was pinned to keep the membrane anchored. Third, only the inward membrane bending (*i.e*., toward the inside of the presynaptic neuron) was allowed. This constraint is in part due to the closely juxtaposed postsynaptic membrane at the synapse that likely prevents the outward membrane bending of the presynaptic terminal. However, other factors may also contribute to this constraint (*e.g*., the asymmetry of lipid distribution within the presynaptic membrane and the presence of pre-localized endocytic proteins).

We leveraged adaptive timestep to simulate the membrane shape changes, in which the membrane is implemented as a union of meshes^99^. Due to the conservation of total membrane area, as some meshes grow during membrane shape changes, some meshes will inevitably shrink. Since the lower bound of timestep in our adaptive simulation scheme positively correlates to the smallest mesh size in the system to ensure the numerical stability, the averaged timestep decreases (Supplementary Fig. 3c), as the simulation converges with the energy minimization and the system reaching the steady state (Supplementary Fig. 3d).

### Reporting summary

Further information on research design is available in the Nature Portfolio Reporting Summary linked to this article.

## Supplementary information

Supplementary Information

Description of additional supplementary files

Supplementary Movie 1

Supplementary Movie 2

Supplementary Movie 3

Supplementary Movie 4

Supplementary Movie 5

Supplementary Movie 6

Reporting Summary

## Data Availability

The additional image data generated in this study have been deposited in the Figshare database under https://figshare.com/projects/Membrane_compression_by_synaptic_vesicle_exocytosis_triggers_ultrafast_endocytosis/166313. Source data are published with the manuscript. Full datasets are available upon request. Source data are provided with this paper.

## Source data

Source data

## Code availability

Custom R, Matlab and Fiji scripts for electron microscopy analysis are available at https://github.com/shigekiwatanabe/SynapsEM. Modeling scripts are available at https://github.com/jliu187/membrane-compression-model. More details can be found in ref. ^102^.
