# Supplementary material for: Membrane compression by synaptic vesicle exocytosis triggers ultrafast endocytosis
Source: Nat Commun. 2023 May 20;14:2888. doi: 10.1038/s41467-023-38595-2 (PMC10199930; doi:10.1038/s41467-023-38595-2)
Supplement: Supplementary file 1 — Supplementary Information [file 41467_2023_38595_MOESM1_ESM.pdf]

a

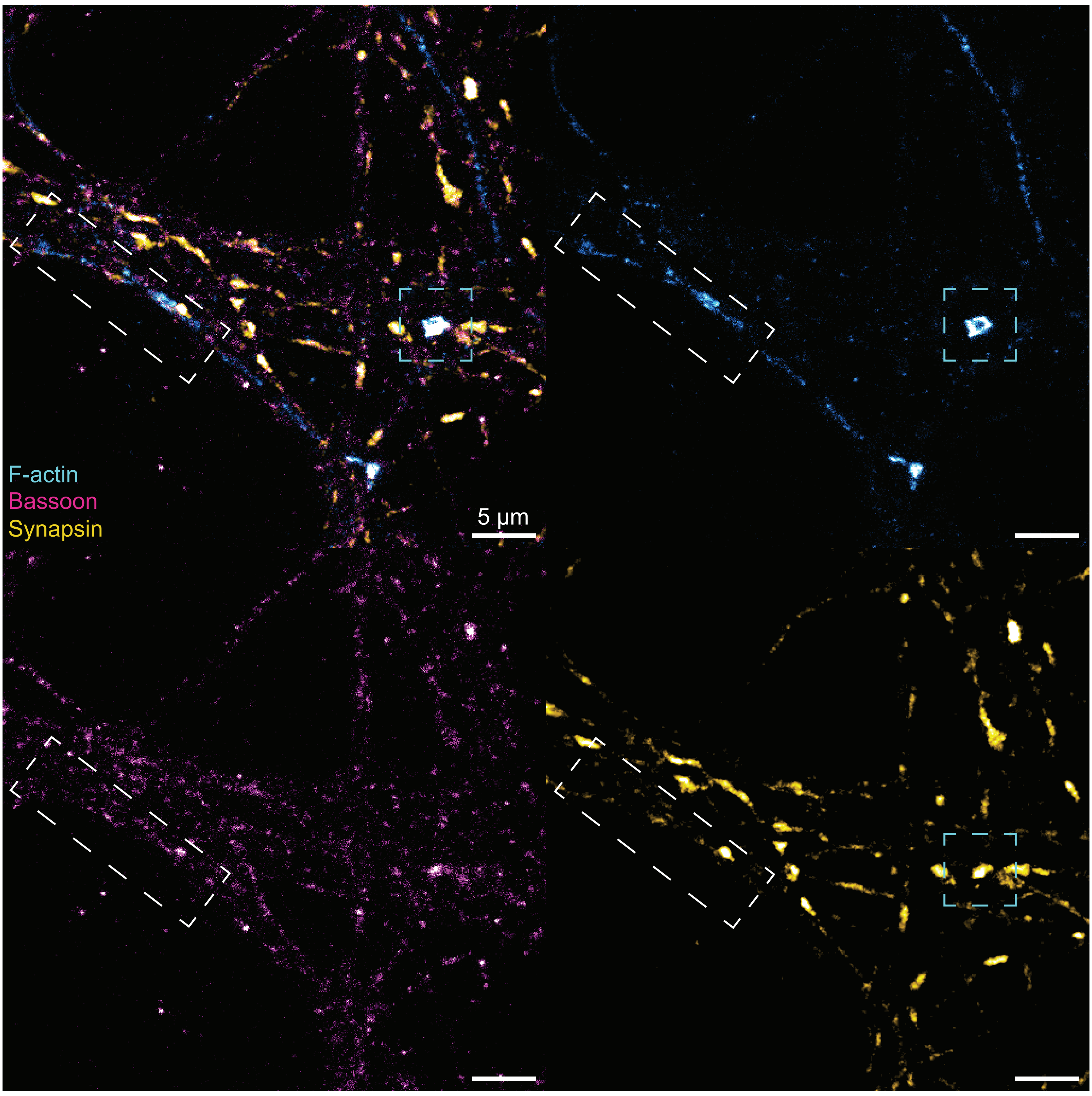
F-actin
Bassoon
Synapsin
5 µm

b

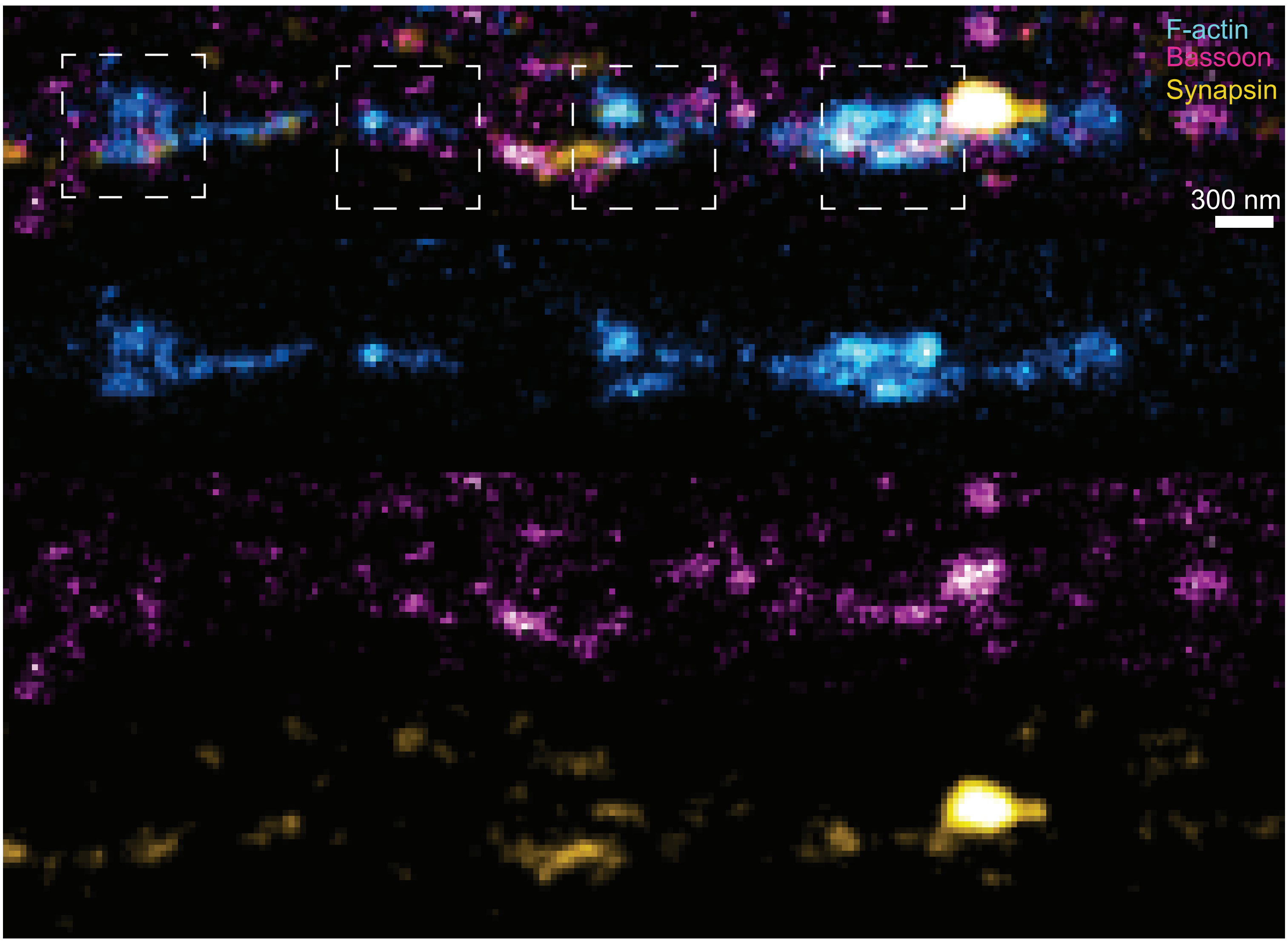
F-actin
Bassoon
Synapsin
300 nm

Supplementary Figure 1.

a. Enlarged field of view of an EGFP-UtrCH (cyan) expressing neuron visualized by 3D gSTED. Image is max projected. Neurons are co-stained with Bassoon (magenta) and Synapsin (yellow). The staining procedure is as described in Fig.1. A composite image is followed by separated channels. The cyan dashed-line box contains the representative presynapse used for Fig.1. The white dashed-line box contains a typical axon.

b. Enlarged view of the axon contained within the dashed-line box in a. Multiple actin ring containing boutons and some partial ring containing boutons are shown, emphasized by white dashed-line boxes. A composite image is followed by separated channels.

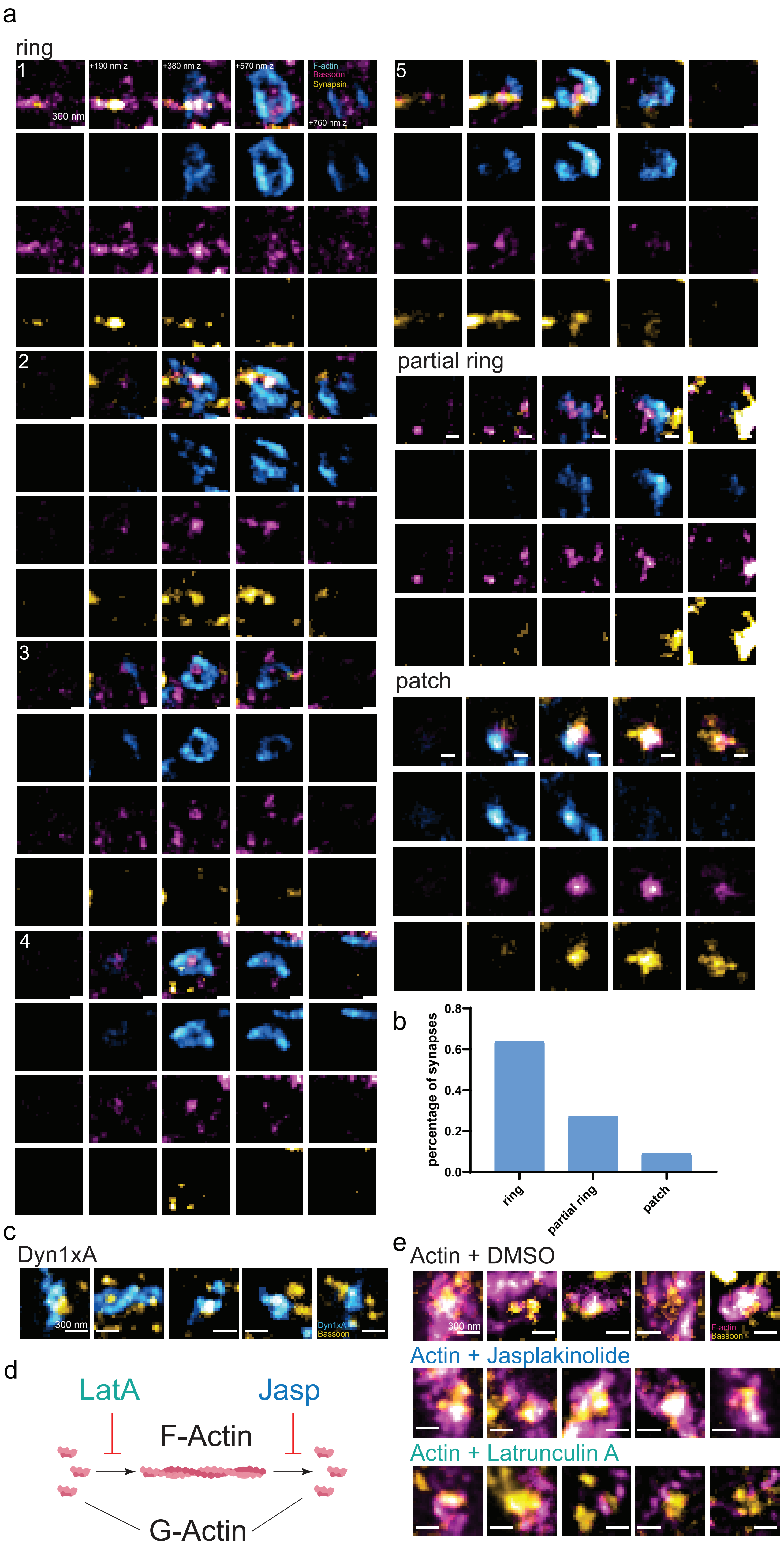

a
ring
1
+190 nm z
+380 nm z
+570 nm z
F-actin
Bassoon
Synapsin
300 nm
+760 nm z
2
3
4
5
partial ring
patch
b
percentage of synapses
0.0
0.2
0.4
0.6
0.8
ring
partial ring
patch
c
Dyn1xA
300 nm
Dyn1xA
Bassoon
d
LatA
Jasp
F-Actin
G-Actin
e
Actin + DMSO
300 nm
F-actin
Bassoon
Actin + Jasplakinolide
Actin + Latrunculin A

Supplementary Figure 2

a. Additional example 3D gSTED micrographs showing the localization of filamentous actin relative to the active zone and reserve pool vesicle cluster in neurons. The active zone is marked by anti-Bassoon antibody and its secondary antibody, the reserve pool vesicle cluster is marked by anti-Synapsin antibody and its secondary antibody. Composite images of synapses are shown above the corresponding three individual channels. Five ring-like actin distributions are shown.

b. Percentage of synapses containing ring-like actin (64%), partial actin rings (27%), or actin patches (9%). Distribution from 33 synapses (n) across 2 cultures (N) reconstructed by 3D gSTED.

c. Example 2D STED micrographs showing the localization of Dynamin 1xA (Dyn1xA) relative to the active zone.

d. Schematic of Latrunculin A (LatA) and Jasplakinolide (Jasp) effects on F-actin filaments. Lat A sequesters actin monomers (Globular-actin, G-actin) to affect the rate of polymerization, while Jasp stabilizes F-actin.

e. Additional example 2D STED micrographs showing the localization of filamentous actin (F-actin) relative to the active zone in neurons treated with DMSO (control), Latrunculin A (Lat A), and Jasplakinolide (Jasp). The active zone is marked by anti-Bassoon antibody and its secondary antibody. (bottom) Effect on actin filaments (F-actin) by Lat A or Jasp treatment.

a

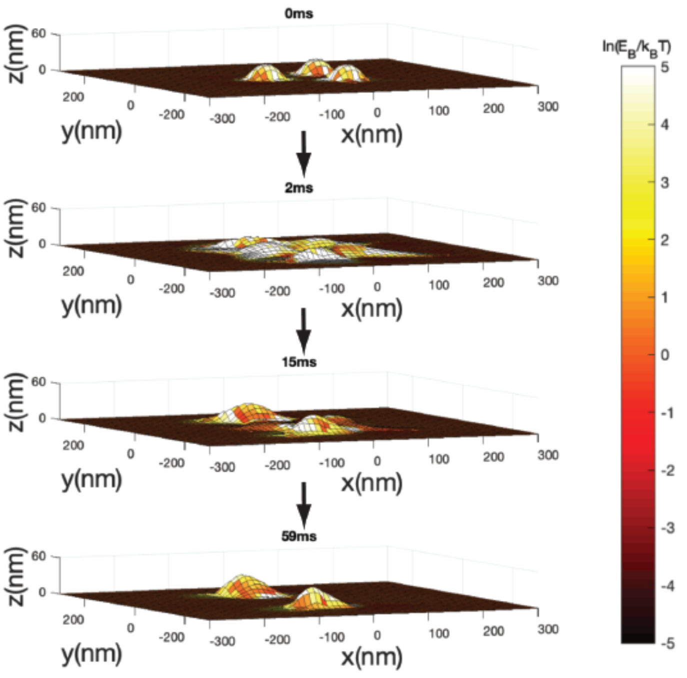
0ms
2ms
15ms
59ms
z(nm)
y(nm)
x(nm)
ln(E_B/k_BT)

b

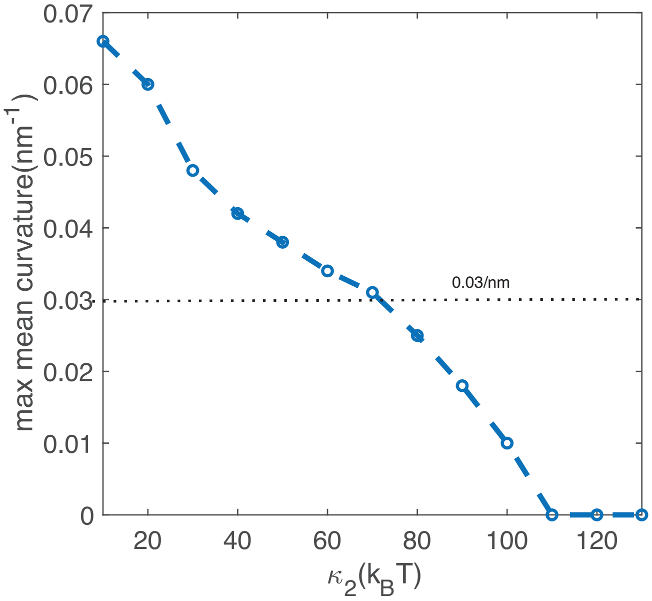
max mean curvature(nm^-1)
κ_2(k_BT)
0.03/nm

c

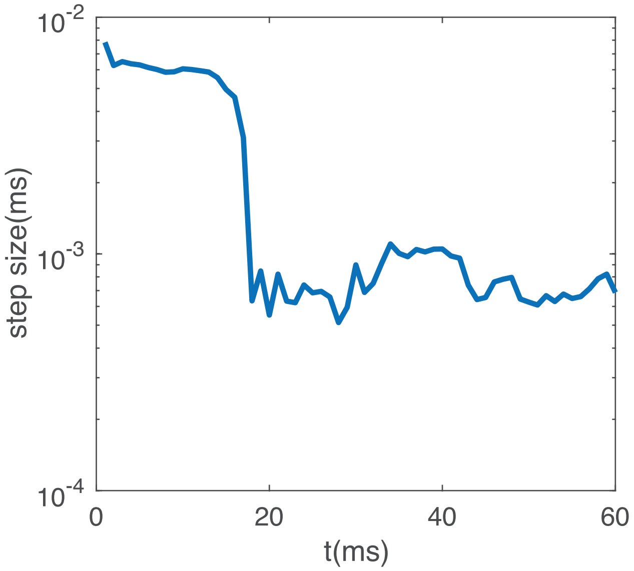
step size(ms)
t(ms)

d

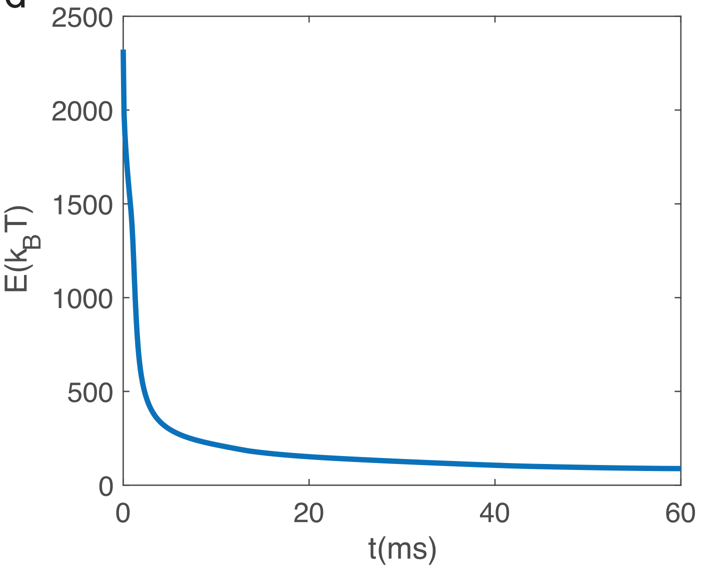
E(k_BT)
t(ms)

Supplementary Figure 3.

a. Snapshots from model simulation showing the spatial-temporal changes in the bending energy (E_B) of the membrane over time. The model parameter and setup correspond to the same condition as the nominal case in Fig. 1e. In the colormap, the bending energy of the local membrane (in the unit of $k_B T$) is plotted in the natural log-scale to optimally discern the spatial-temporal changes.

b. Model results showing that the maximal mean curvature of the resulting endocytic pits decreases with the bending modulus of the active zone and endocytic zone, κ_2, while keeping all the other parameters and the geometry of fusing vesicles as those in the nominal case in Fig. 1e.

c. A representative trajectory showing the evolution of the adaptive simulation timestep over time in the numerical simulation of the nominal case in Fig. 1e.

d. Model result showing that the total energy of the system is minimized over time during the simulation. Here, the model parameter and setup correspond to the same condition as the nominal case in Fig. 1e.

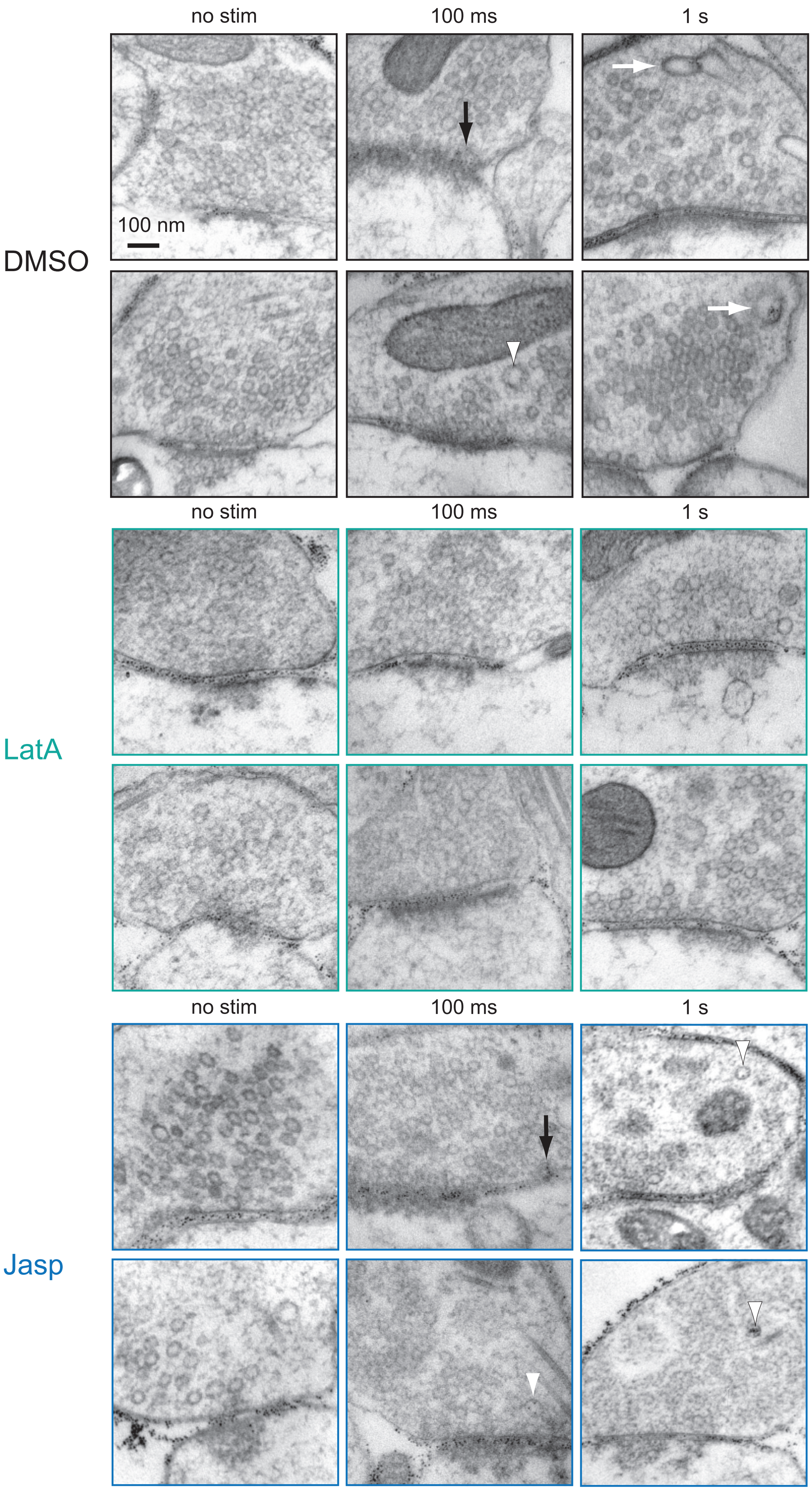

no stim
100 ms
1 s
100 nm
DMSO
LatA
Jasp

Supplementary Figure 4.

Additional electron micrographs showing ChetaTC-expressing wild-type neurons, treated with 0.1% DMSO, 10 µM Latrunculin A (LatA), and 100 nM Jasplakinolide. The left panel show unstimulated conditions, while the right panel show 100 ms after single stimulus (10 ms light pulse, 37 ºC, 4 mM external Ca^{2+}). Black arrow: endocytic pit. White arrowhead: ferritin-positive endocytic vesicle. White arrow: ferritin-positive endosomes. N=2 cultures, n=200.

a

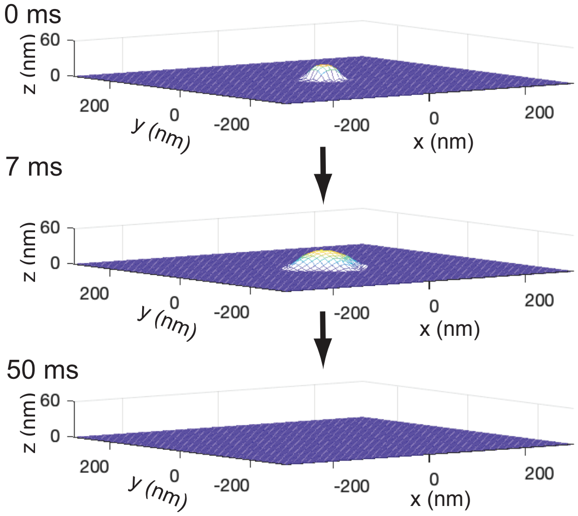
0 ms
z (nm)
60
0
200
0
-200
y (nm)
-200
0
200
x (nm)
7 ms
z (nm)
60
0
200
0
-200
y (nm)
-200
0
200
x (nm)
50 ms
z (nm)
60
0
200
0
-200
y (nm)
-200
0
200
x (nm)

Supplementary Figure 5.

a. Snapshots from simulation, showing the evolution of membrane shape within the active zone upon a single vesicle fusion. As long as the membrane area is conserved, the fusing vesicle cannot flatten out completely. However, when the membrane area conservation was turned off at 7 ms, the exocytic pit fully collapse into the active zone.

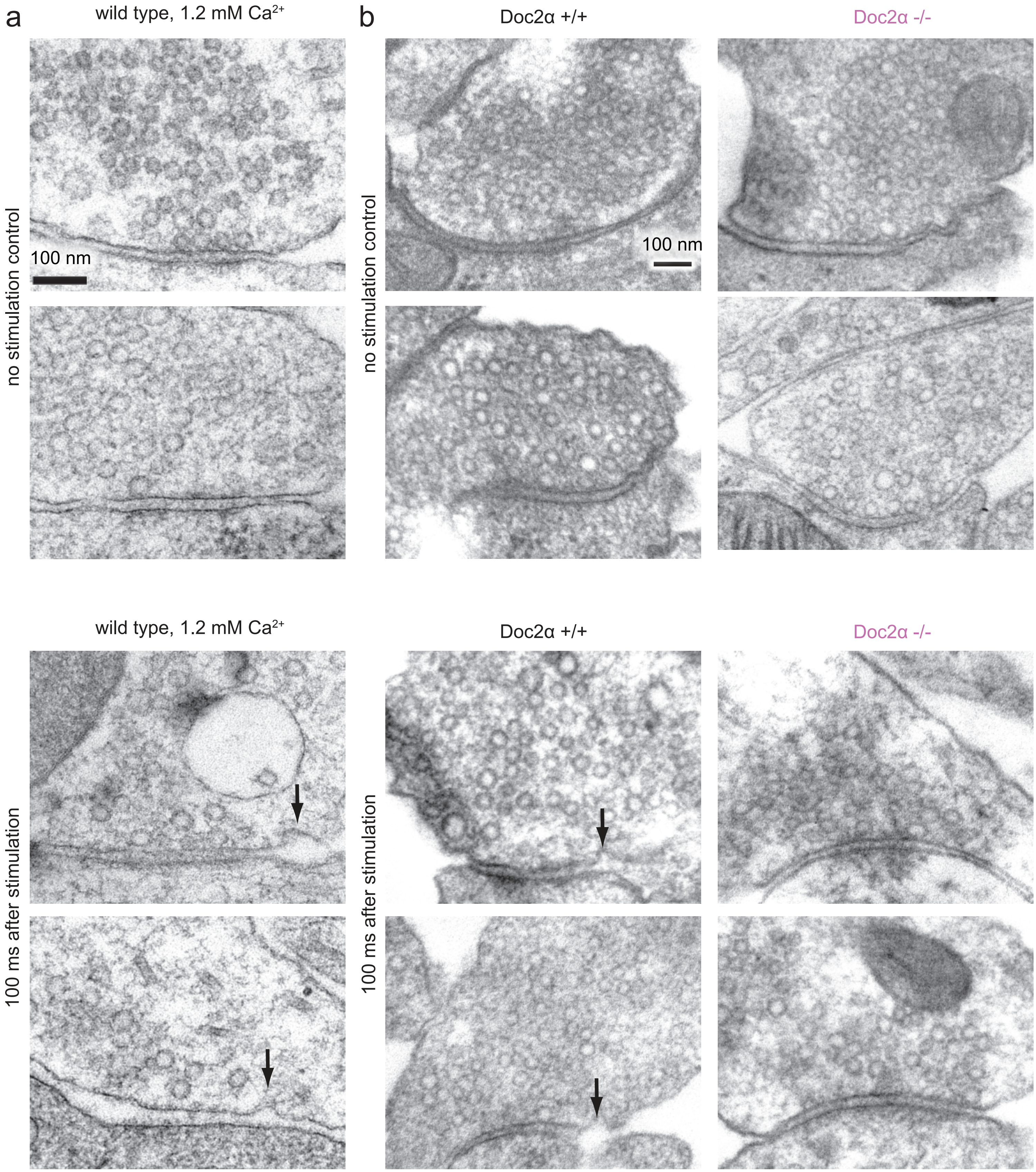
a
wild type, 1.2 mM Ca2+
no stimulation control
100 nm
b
Doc2α +/+
Doc2α -/-
no stimulation control
100 nm
wild type, 1.2 mM Ca2+
100 ms after stimulation
Doc2α +/+
Doc2α -/-
100 ms after stimulation

Supplementary Figure 6.

a. Additional example micrographs showing wild-type synapses unstimulated (left) or stimulated with electric field for 1 ms and frozen 100 ms later (right). The external calcium concentration is 1.2 mM. Black arrow: endocytic pit. N=2 cultures, n=200.

b. Additional example micrographs showing wild-type and Doc2α knockout unstimulated or stimulated with electric field for 1 ms and frozen 100 ms later. Black arro[illegible] endocytic pit. N=2 cultures, n=200.

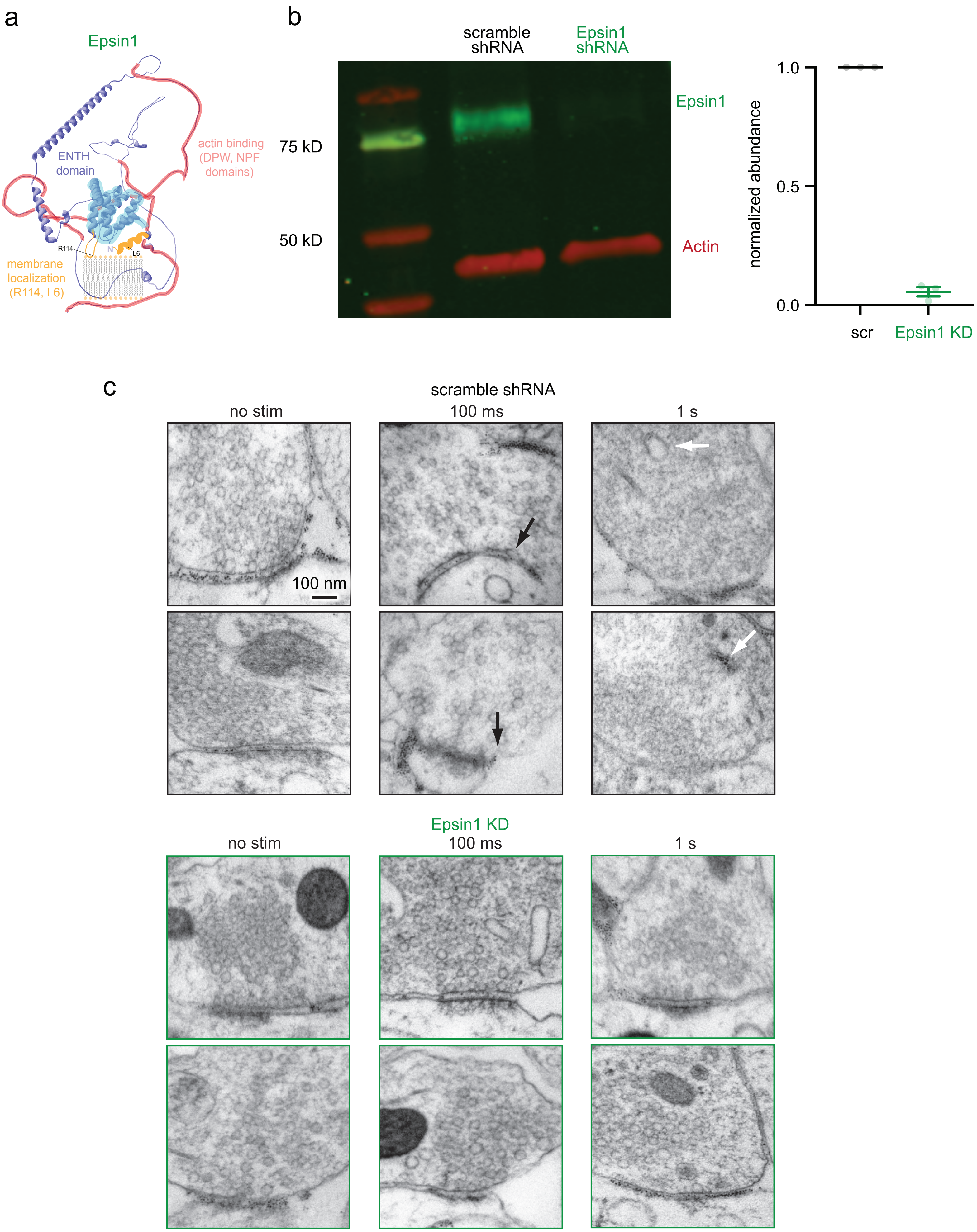

a
Epsin1
ENTH domain
actin binding (DPW, NPF domains)
R114
L6
membrane localization (R114, L6)
b
scramble shRNA
Epsin1 shRNA
75 kD
50 kD
Epsin1
Actin
normalized abundance
1.0
0.5
0.0
scr
Epsin1 KD
c
scramble shRNA
no stim
100 ms
1 s
100 nm
Epsin1 KD
no stim
100 ms
1 s

Supplementary Figure 7.

a. A schematic showing the protein structural elements of Epsin1. Epsin1 contains ENTH domain, NPF and DPW. The C-terminal domain, marked light red, interacts with F-actin, while the ENTH domain, marked light blue, interacts with plasma membrane.

b. An example Western blot and a plot showing efficiency of Epsin1 knock-down (KD). In each case, Epsin1 signal intensity is normalized by the amount of actin, and then, the abundance of Epsin1 is calculated based on the normalized intensity in the scramble (scr) shRNA control. The mean and SEM are shown. Dot: one culture. N=3 cultures, ***$p<0.001$.

c. Additional example electron micrographs showing wild-type and Epsin1 KD s unstimulated or stimulated with a single electrical pulse (1 ms) and frozen 100 ms or 1 s later. Black arrow: endocytic pit. White arrow: ferritin-positive endosomes.

Supplementary Table 1: Model Parameters used in this study.

**Model Parameter**	**Physical Range**	**Nominal Value used in the model**
Bending modulus of post-fusion vesicle, κ_1	100 – 400 k_BT (~ 4.3×10^{-19} J – 1.7×10^{-18} J)[31,33]	300 k_BT (~ 1.3×10^{-18} J)
Bending modulus of the active zone and endocytic zone, κ_2	10 – 100 k_BT (~ 4.3×10^{-20} J – 4.3×10^{-19} J)[40,41]	20 k_BT (~8.6×10^{-20} J)
Bending modulus of the actin-enriched periactive zone, κ_3	Same as κ_1 [42–44]	300 k_BT (~ 1.3×10^{-18} J)
Osmotic pressure	~ 1 kPa [103]	1 kPa
Effective viscous drag coefficient of membrane shape changes	~ 2×10^9 Pa·s·m^{-1} [104]	2×10^9 Pa·s·m^{-1}

Note (*): The temperature, T, is chosen to be 37°C.

Supplementary Table 2: A summary of statistical analysis used in this study.

Fig. 1b distribution

	DMSO	Jasp	LatA	Dyn1xA
Test for normal distribution				
Anderson-Darling test				
A2*	0.4894	0.3809	0.3179	1.018
P value	0.1783	0.3423	0.4913	0.0071
Passed normality test (alpha=0.05)?	Yes	Yes	Yes	No
P value summary	ns	ns	ns	**
D'Agostino & Pearson test				
K2	3.607	3.721	3.097	2.671
P value	0.1647	0.1556	0.2126	0.263
Passed normality test (alpha=0.05)?	Yes	Yes	Yes	Yes
P value summary	ns	ns	ns	ns
Shapiro-Wilk test				
W	0.8899	0.9075	0.9234	0.7993
P value	0.1175	0.1982	0.315	0.0092
Passed normality test (alpha=0.05)?	Yes	Yes	Yes	No
P value summary	ns	ns	ns	**
Kolmogorov-Smirnov test				
KS distance	0.1736	0.141	0.1223	0.2417
P value	>0.1000	>0.1000	>0.1000	0.0511
Passed normality test (alpha=0.05)?	Yes	Yes	Yes	Yes
P value summary	ns	ns	ns	ns
Number of values (n)	339	390	458	185
Number of cultures (N)	3	3	3	2
Minimum	-33.38	-14.86	-16.63	-27.97
25% Percentile	95.2	132.2	114.5	61.09
Median	169.4	218.4	212.5	114.1
75% Percentile	262.9	326	349.8	167.7
Maximum	514	545.6	544.5	440.6
Range	547.3	560.5	561.1	468.5
Mean	187.4	233	234.4	122.1
Std. Deviation	122.4	131.7	141.9	89.97
Std. Error of Mean	6.647	6.668	6.631	6.615

Dunn's multiple comparisons test	Mean rank diff.	Significant?	Summary	Adjusted P Value
DMSO vs. Jasp	-131.3	Yes	****	<0.0001
DMSO vs. LatA	-124.7	Yes	****	<0.0001
DMSO vs. Dyn1xA	208	Yes	****	<0.0001
Jasp vs. LatA	6.588	No	ns	>0.9999
Jasp vs. Dyn1xA	339.3	Yes	****	<0.0001
LatA vs. Dyn1xA	332.7	Yes	****	<0.0001

Fig. 1b intensity

	DMSO	Jasp	LatA	
Test for normal distribution				
Anderson-Darling test				
A2*	2.222	2.167	2.618	
P value	<0.0001	<0.0001	<0.0001	
Passed normality test (alpha=0.05)?	No	No	No	
P value summary	****	****	****	
D'Agostino & Pearson test				
K2	4.986	5.498	11.76	
P value	0.0826	0.064	0.0028	
Passed normality test (alpha=0.05)?	Yes	Yes	No	
P value summary	ns	ns	**	
Shapiro-Wilk test				
W	0.7445	0.75	0.6975	
P value	0.0001	0.0001	<0.0001	
Passed normality test (alpha=0.05)?	No	No	No	
P value summary	***	***	****	
Kolmogorov-Smirnov test				
KS distance	0.2435	0.2688	0.2651	
P value	0.0021	0.0004	0.0005	
Passed normality test (alpha=0.05)?	No	No	No	
P value summary	**	***	***	
Number of values (n)	134	179	233	
Number of cultures (N)	3	3	3	
Minimum	0.5	2.416	0	

25% Percentile	7.244	6.718	4.798	
Median	9.921	9.592	6.847	
75% Percentile	14.51	14.24	11.06	
Maximum	34.02	40	32.9	
Range	33.52	37.59	32.9	
Mean	11.33	11.33	8.556	
Std. Deviation	5.647	6.298	5.588	
Std. Error of Mean	0.4878	0.4707	0.3661	
Kruskal-Wallis test				
Dunn's multiple comparisons test	Mean rank diff.	Significant?	Summary	Adjusted P Value
DMSO vs. Jasp	7.691	No	ns	>0.9999
DMSO vs. LatA	94.14	Yes	****	<0.0001
Jasp vs. LatA	86.45	Yes	****	<0.0001

Fig. 2d Kruskal-Wallis test with Dunn's multiple comparisons test

		N (culture)	n (synatic profiles)	mean	sem	p values (against no stim control)
DMSO	no stim	2	200	0.01	0.007053	
	100 ms	2	200	0.105	0.02173	0.0002
LatA	no stim	2	200	0	0	
	100 ms	2	200	0.005	0.005	>0.9999
Jasp	no stim	2	193	0.00518	0.005181	
	100 ms	2	200	0.17	0.02663	<0.0001

Dunn's multiple comparisons test	Mean rank diff.	Significant?	Summary	Adjusted P Value
DMSO no stim vs. DMSO 100 ms	-56.67	Yes	***	0.0002
DMSO no stim vs. LatA no stim	5.965	No	ns	>0.9999
DMSO no stim vs. LatA 100 ms	2.983	No	ns	>0.9999
DMSO no stim vs. Jasp no stim	2.874	No	ns	>0.9999
DMSO no stim vs. Jasp 100 ms	-95.44	Yes	****	<0.0001
DMSO 100 ms vs. LatA no stim	62.63	Yes	****	<0.0001
DMSO 100 ms vs. LatA 100 ms	59.65	Yes	****	<0.0001
DMSO 100 ms vs. Jasp no stim	59.54	Yes	****	<0.0001

DMSO 100 ms vs. Jasp 100 ms	-38.77	Yes	*	0.0409
LatA no stim vs. LatA 100 ms	-2.982	No	ns	>0.9999
LatA no stim vs. Jasp no stim	-3.091	No	ns	>0.9999
LatA no stim vs. Jasp 100 ms	-101.4	Yes	****	<0.0001
LatA 100 ms vs. Jasp no stim	-0.1082	No	ns	>0.9999
LatA 100 ms vs. Jasp 100 ms	-98.42	Yes	****	<0.0001
Jasp no stim vs. Jasp 100 ms	-98.31	Yes	****	<0.0001

Fig. 2e Kruskal-Wallis test with Dunn's multiple comparisons test

		N (culture)	n (synatic profiles)	mean	sem	p values (against no stim control)
DMSO	no stim	2	237	0.09705	0.02188	
	1 s	2	207	0.2222	0.0313	0.0009
LatA	no stim	2	207	0.01932	0.009591	
	1 s	2	209	0.01914	0.0095	>0.9999
Jasp	no stim	2	199	0.05528	0.01624	
	1 s	2	213	0.1784	0.03021	0.0097

Dunn's multiple comparisons test	Mean rank diff.	Significant?	Summary	Adjusted P Value
DMSO no stim vs. DMSO 1s	-75.92	Yes	***	0.0002

DMSO no stim vs. LatA no stim	44.08	No	ns	0.1764
DMSO no stim vs. LatA 1s	44.2	No	ns	0.1699
DMSO no stim vs. Jasp no stim	21.34	No	ns	>0.9999
DMSO no stim vs. Jasp 1 s	-45.49	No	ns	0.1321
DMSO 1s vs. LatA no stim	120	Yes	****	<0.0001
DMSO 1s vs. LatA 1s	120.1	Yes	****	<0.0001
DMSO 1s vs. Jasp no stim	97.26	Yes	****	<0.0001
DMSO 1s vs. Jasp 1 s	30.43	No	ns	>0.9999
LatA no stim vs. LatA 1s	0.117	No	ns	>0.9999
LatA no stim vs. Jasp no stim	-22.74	No	ns	>0.9999
LatA no stim vs. Jasp 1 s	-89.57	Yes	****	<0.0001
LatA 1s vs. Jasp no stim	-22.86	No	ns	>0.9999
LatA 1s vs. Jasp 1 s	-89.68	Yes	****	<0.0001
Jasp no stim vs. Jasp 1 s	-66.83	Yes	**	0.0034

Fig. 3d Mann-Whitney test

		N (culture)	n (synatic profiles)	mean	sem	p values (against no stim control)
wild type	no stim	2	209	0.00479	0.004785	
	100 ms	2	218	0.1147	0.0235	<0.0001

Fig. 3f Kruskal-Wallis test with Dunn's correction

		N (culture)	n (synatic profiles)	mean	sem	p values (against no stim control)
Doc2a+/+	no stim	2	243	0.02881	0.01075	
	100 ms	2	212	0.1274	0.0239	0.0012
Doc2a-/-	no stim	2	235	0.04681	0.01381	
	100 ms	2	234	0.03846	0.01398	0.9742

Dunn's multiple comparisons test	Mean rank diff.	Significant?	Summary	Adjusted P Value
wt no stim vs. wt 100 ms	-43.38	Yes	****	<0.0001
wt no stim vs. doc2a no stim	-8.299	No	ns	>0.9999
wt no stim vs. doc2a 100 ms	-2.592	No	ns	>0.9999
wt 100 ms vs. doc2a no stim	35.08	Yes	**	0.003
wt 100 ms vs. doc2a 100 ms	40.79	Yes	***	0.0003
doc2a no stim vs. doc2a 100 ms	5.707	No	ns	>0.9999

Fig. 3h Kruskal-Wallis test with Dunn's correction

		N (culture)	n (synatic profiles)	mean	sem	p values (against no stim control)
DMSO	no stim	2	200	0.025	0.01107	
	100 ms	2	167	0.1078	0.0269	0.0012
EGTA	no stim	2	201	0	0	
	100 ms	2	159	0.02516	0.01246	>0.9999

Dunn's multiple comparisons test	Mean rank diff.	Significant?	Summary	Adjusted P Value

no stim vs. 100 ms	-25.82	Yes	**	0.0012
no stim vs. no stim	9.063	No	ns	>0.9999
no stim vs. 100 ms	-0.057	No	ns	>0.9999
100 ms vs. no stim	34.88	Yes	****	<0.0001
100 ms vs. 100 ms	25.76	Yes	**	0.0027
no stim vs. 100 ms	-9.119	No	ns	>0.9999

Fig. 4e Kruskal-Wallis test with Dunn's correction

	N (culture)	n (synapse)	minimum	25%	median	75%	maximum	skewness
scramble	3	75	108.3	219.2	374.8	661.9	1269	0.7593
Epsin1 KD	3	66	62.49	123.8	221.4	376.5	1324	1.868

Fig. 4g Games-Howell's multiple comparisons test

		N (culture)	n (synatic profiles)	mean	sem	p values (against no stim control)
scramble	no stim	2	225	0.02222	0.00985	
	100 ms	2	118	0.1356	0.03165	0.0045
Epsin1 KD	no stim	2	235	0.00426	0.00426	
	100 ms	2	199	0.01005	0.00709	0.8967

Games-Howell's multiple comparisons test	Mean Diff.	95.00% CI of diff.	Below threshold?	Summary	Adjusted P Value
scr no stim vs. scr 100 ms	-0.1134	-0.1996 to -0.0271	Yes	**	0.0045
scr no stim vs. kd no stim	0.01797	-0.009748 to	No	ns	0.3389
scr no stim vs. kd 100 ms	0.01217	-0.01914 to	No	ns	0.7477
scr 100 ms vs. kd no stim	0.1313	0.04815 to 0.2145	Yes	***	0.0004
scr 100 ms vs. kd 100 ms	0.1255	0.04112 to 0.2100	Yes	***	0.001
kd no stim vs. kd 100 ms	-0.005795	-0.02714 to	No	ns	0.8967

Fig. 4h Games-Howell's multiple comparisons test

		N (culture)	n (synatic profiles)	mean	sem	p values (against no stim control)
scramble	no stim	2	225	0.03556	0.01237	

	1 s	2	127	0.3071	0.04543	<0.0001
Epsin1 KD	no stim	2	235	0.08085	0.02152	
	1 s	2	232	0.0819	0.02	>0.9999

Games-Howell's multiple comparisons test	Mean Diff.	95.00% CI of diff.	Below threshold?	Summary	Adjusted P Value
scr no stim vs. scr 1 s	-0.2715	-0.3939 to -	Yes	****	<0.0001
scr no stim vs. kd no stim	-0.0453	-0.1094 to 0.0187	No	ns	0.2633
scr no stim vs. kd 1 s	-0.04634	-0.1070 to	No	ns	0.2011
scr 1 s vs. kd no stim	0.2262	0.09591 to	Yes	****	<0.0001
scr 1 s vs. kd 1 s	0.2252	0.09644 to	Yes	****	<0.0001
kd no stim vs. kd 1 s	-0.001045	-0.0768	No	ns	>0.9999
