# Supplementary material for: Membrane compression by synaptic vesicle exocytosis triggers ultrafast endocytosis
Source: Nat Commun. 2023 May 20;14:2888. doi: 10.1038/s41467-023-38595-2 (PMC10199930; doi:10.1038/s41467-023-38595-2)
Supplement: Supplementary file 2 — Description of additional supplementary files [file 41467_2023_38595_MOESM2_ESM.pdf]

Description of additional Supplementary files

Supplementary Movie 1 : Movie related to Fig. 1e. The spatial-temporal evolution of membrane bending energy (log scale) corresponding to figure 1e.

Supplementary Movie 2 : Movie related to Fig. 1e, but showing evolutions of the local membrane bending energy. The membrane is represented by the mesh, and the colormap gauges the bending energy of the local membrane (in the unit of kBT), which is plotted in the natural log-scale to optimally discern the spatial-temporal changes.

Supplementary Movie 3 : Movie related to Fig. 2a, showing the evolution of membrane curvature within the active zone by fusion of three vesicles when the membrane area conservation is initially on but turned off at 5 ms.

Supplementary Movie 4 : Movie related to Fig. 3i, showing the evolution of membrane curvature within the active zone when the second vesicle is fused when the depth of first vesicle is at 10 nm.

Supplementary Movie 5 : Movie related to Fig. 3j, showing the evolution of membrane curvature within the active zone when the second vesicle is fused when the depth of first vesicle is at 1 nm.

Supplementary Movie 6 : Movie related to Fig. 3k, showing the evolution of membrane curvature within the active zone when the second vesicle is fused much later, after the first vesicle completely flattens out by relaxing the membrane area conservation.
