# Supplementary material for: Membrane compression by synaptic vesicle exocytosis triggers ultrafast endocytosis
Source: Nat Commun. 2023 May 20;14:2888. doi: 10.1038/s41467-023-38595-2 (PMC10199930; doi:10.1038/s41467-023-38595-2)
Supplement: Supplementary file 9 — Reporting Summary [file 41467_2023_38595_MOESM9_ESM.pdf]

nature portfolio

Corresponding author(s): Shigeki Watanabe, Jian Liu

Last updated by author(s): 5/3/2023

Reporting Summary

Nature Portfolio wishes to improve the reproducibility of the work that we publish. This form provides structure for consistency and transparency in reporting. For further information on Nature Portfolio policies, see our Editorial Policies and the Editorial Policy Checklist.

Statistics

For all statistical analyses, confirm that the following items are present in the figure legend, table legend, main text, or Methods section.

n/a	Confirmed	
☐	☒	The exact sample size (*n*) for each experimental group/condition, given as a discrete number and unit of measurement
☐	☒	A statement on whether measurements were taken from distinct samples or whether the same sample was measured repeatedly
☐	☒	The statistical test(s) used AND whether they are one- or two-sided<br>*Only common tests should be described solely by name; describe more complex techniques in the Methods section.*
☐	☒	A description of all covariates tested
☐	☒	A description of any assumptions or corrections, such as tests of normality and adjustment for multiple comparisons
☐	☒	A full description of the statistical parameters including central tendency (e.g. means) or other basic estimates (e.g. regression coefficient) AND variation (e.g. standard deviation) or associated estimates of uncertainty (e.g. confidence intervals)
☐	☒	For null hypothesis testing, the test statistic (e.g. *F*, *t*, *r*) with confidence intervals, effect sizes, degrees of freedom and *P* value noted<br>*Give P values as exact values whenever suitable.*
☒	☐	For Bayesian analysis, information on the choice of priors and Markov chain Monte Carlo settings
☒	☐	For hierarchical and complex designs, identification of the appropriate level for tests and full reporting of outcomes
☐	☒	Estimates of effect sizes (e.g. Cohen's *d*, Pearson's *r*), indicating how they were calculated

Our web collection on statistics for biologists contains articles on many of the points above.

Software and code

Policy information about availability of computer code

Data collection	AMT Capture v6 for electron microscopy data acquisition. Imspector data acquisition program for STED imaging (v0.05), Zen v2.3SP1 for Airyscan imaging. Surface Evolver v2.70
Data analysis	SynapsEM analysis codes for electron microscopy analysis (https://github.com/shigekiwatanabe/SynapsEM), Custom analysis scripts for Matlab (v2015 or older), Custom modeling scripts (https://github.com/jliu187/membrane-compression-model), GraphPad Prism 7 or older, ImageJ.

For manuscripts utilizing custom algorithms or software that are central to the research but not yet described in published literature, software must be made available to editors and reviewers. We strongly encourage code deposition in a community repository (e.g. GitHub). See the Nature Portfolio guidelines for submitting code & software for further information.

Data

Policy information about availability of data

All manuscripts must include a data availability statement. This statement should provide the following information, where applicable:

- Accession codes, unique identifiers, or web links for publicly available datasets
- A description of any restrictions on data availability
- For clinical datasets or third party data, please ensure that the statement adheres to our policy

The additional image data generated in this study have been deposited in the Figshare database https://figshare.com/projects/

Membrane_compression_by_synaptic_vesicle_exocytosis_triggers_ultrafast_endocytosis/166313 The XX data generated in this study are provided in the Supplementary Information/Source Data file. Source data are published with the manuscript. Full datasets are available upon request.

Human research participants

Policy information about studies involving human research participants and Sex and Gender in Research.

Reporting on sex and gender	N/A
Population characteristics	N/A
Recruitment	N/A
Ethics oversight	*Identify the organization(s) that approved the study protocol.*

Note that full information on the approval of the study protocol must also be provided in the manuscript.

Field-specific reporting

Please select the one below that is the best fit for your research. If you are not sure, read the appropriate sections before making your selection.

☒ Life sciences ☐ Behavioural & social sciences ☐ Ecological, evolutionary & environmental sciences

For a reference copy of the document with all sections, see nature.com/documents/nr-reporting-summary-flat.pdf

Life sciences study design

All studies must disclose on these points even when the disclosure is negative.

Sample size	Power analysis was not used to determine the number (n) of synaptic profiles (2D EM). Our threshold of n >200 (from N = 2 or more experiments) for synaptic profiles was taken from previous work (Watanabe et al., 2013), based on 15-20% of synapses containing endocytic or exocytic events, such that >30 synapses with endocytic or exocytic events would be captured. For fluorescence imaging, n>30 synaptic ROI's (from N=2 independent cultures) were chosen for each experiment. ~30 synaptic ROIs is based on previous work which suggests differences between groups (intensity, distribution, frequency, number etc.) are measured with ~30 synapses.
Data exclusions	Images that could not be reliably segmented, either because the image was not of a bona fide synapse or morphology was too poor, were excluded from segmentation; this was done only after randomizing the images. No other data were excluded.
Replication	All experiments were performed at least twice (from separate litters, different rounds of neuronal cell culture, frozen and processed separately, and segmented in separate batches of randomized images). Similar results were obtained in each experiment.
Randomization	No randomization of experimental groups was performed prior to freezing. Experimental conditions are not sensitive to biological conditions (e.g. control vs. knockdown). For image segmentations, images were always randomized before manual segmentation.
Blinding	To limit bias, synapses were found by bidirectional raster scanning along the section at 100,000x, which makes it difficult to "pick" certain synapses, as a synapse usually takes up most of this field of view, and anything that appeared to be a synapse was imaged without close examination. For fluorescence imaging, samples are scanned until identifying transfected cells, from which point all synapses visible are collected as ROIs for analysis, reducing bias similarly to electron microscopy analysis. In all cases, experimentalists were blinded to sample conditions (genotypes, drug treatment, time points, etc.) during the image acquisition and analysis.

Reporting for specific materials, systems and methods

We require information from authors about some types of materials, experimental systems and methods used in many studies. Here, indicate whether each material, system or method listed is relevant to your study. If you are not sure if a list item applies to your research, read the appropriate section before selecting a response.

Materials & experimental systems

n/a	Involved in the study
☐	☒ Antibodies
☐	☒ Eukaryotic cell lines
☒	☐ Palaeontology and archaeology
☐	☒ Animals and other organisms
☒	☐ Clinical data
☒	☐ Dual use research of concern

Methods

n/a	Involved in the study
☒	☐ ChIP-seq
☒	☐ Flow cytometry
☒	☐ MRI-based neuroimaging

Antibodies

Antibodies used

Anti-Epsinl Rabbit Polyclonal. Thermo Fisher, Cat no PAS-44242
Anti Bassoon Mouse Monoclonal Synaptic System Cat no 141 011
Anti-synapsin 1/2 Polyclonal Guinea Pig synaptic systems Cat no 106 004
Anti-GFP Rabbit Polyclonal MBL International, Cat no 598
Goat Anti-Mouse IgG Polyclonal Antibody (IRDye® 680RD), LI-COR Biosciences Cat no 925-68070
Goat Anti-Rabbit IgG Polyclonal Antibody (IRDye® 800CW), LI-COR Biosciences Cat no 925-32211

Validation

Anti-Epsinl antibody specificity towards mouse Epsinl was validated by knocking down Epsinl, reported in the manuscript. Anti Bassoon was reported to react with Rat and Mouse, https://www.sysy.com/product/141011
Anti-synapsin is specific for synapsins 1a/b and 2a/b, K.O. validated, https://sysy.com/product/106004
Anti GFP validation https://www.mblintl.com/products/598/

Eukaryotic cell lines

Policy information about cell lines and Sex and Gender in Research

Cell line source(s)

HEK293T cells were acquired from ATCC.

Authentication

The cell line was not authenticated.

Mycoplasma contamination

The cell line was not tested for mycoplasma contamination.

Commonly misidentified lines
(See ICLAC register)

N/A

Animals and other research organisms

Policy information about studies involving animals; ARRIVE guidelines recommended for reporting animal research, and Sex and Gender in Research

Laboratory animals

E18 or P0 wild-type C57BL/6J and Doc2a mice of both sexes were used for all experiments. The sex of newborn or embryonic pups cannot be identified, but cells for neuronal culture were pooled from all the mice in a litter, and so contained cells from mice of both sexes in each experiment.

Wild animals

No wild animals were used in the study

Reporting on sex

Primary neuronal cultures were prepared from embryonic day 18 (E18) or postnatal day 0 (P0) animals. The sex of newborn or embryonic pups cannot be identified, but cells for neuronal culture were pooled from all the mice in a litter, and so contained cells from mice of both sexes in each experiment.

Field-collected samples

No field collected samples were used in the study.

Ethics oversight

All animal care was performed according to the National Institutes of Health guidelines for animal research with approval from the Animal Care and Use Committee at the Johns Hopkins University School of Medicine.

Note that full information on the approval of the study protocol must also be provided in the manuscript.
